# Sequencing-based high throughput mutation detection in bread wheat

**DOI:** 10.1186/s12864-015-2112-1

**Published:** 2015-11-17

**Authors:** Gaganjot Sidhu, Amita Mohan, Ping Zheng, Amandeep Kaur Dhaliwal, Dorrie Main, Kulvinder S Gill

**Affiliations:** Department of Crop and Soil Sciences, Washington State University, PO Box 646420, Pullman 99164-6420, WA USA; Department of Horticulture, Washington State University, PO Box 646414, Pullman 99164-6414, WA USA

**Keywords:** Ethylmethane sulfonate, Mutation, Genotyping-by-sequencing, Bread wheat, Unigenes, Bioinformatics analysis pipeline, EST mapping

## Abstract

**Background:**

Forward genetic approaches have limited use for agronomic traits that can’t be reliably scored on a single plant basis. Thus, mutants in wheat and other crops are more useful for gene function studies by reverse genetic approach. With a long-term goal to develop a sequence-based mutation detection resource in hexaploid wheat, we conducted a feasibility study to accurately differentiate induced mutations from the homoeologs’ sequence variations present among the three wheat genomes.

**Results:**

A reduced representation *Ape*KI library consisting of 21 Ethylmethane Sulfonate (EMS) induced mutants and two wild type cv. Indian plants was developed using individual barcode adapters and sequenced. A novel bioinformatics pipeline was developed to identify sequence variants using 178,464 wheat unigenes as a reference wheat transcriptome. In total, 14,130 mutational changes [Single Nucleotide Polymorphisms (SNPs) and Insertions/Deletions (INDELs)] and 150,511 homoeologous sequence changes were detected. On an average, 662 SNPs (ranging from 46 to 1,330) and 10 small INDELs (ranging from 0 to 23) were identified for each of the mutants. A mutation frequency of one per 5 Kb was observed with 70 % being transitions and 30 % transversions. The pipeline was tested using the known sequence changes in the three wheat genes. Genes present in the distal regions of the chromosomes were found to be more prone to EMS compared to genes present in the proximal regions. Redefined parameters identified a total of 28,348 mutational changes (1,349/plant).

**Conclusions:**

We conclude that sequencing based mutation detection is a valuable method to identify induced mutations at large.

**Electronic supplementary material:**

The online version of this article (doi:10.1186/s12864-015-2112-1) contains supplementary material, which is available to authorized users.

## Background

The development of functional genomic resources in wheat is critical for determining function, especially for genes controlling agronomic traits. The creation of such resources involves disruption of genes either by mutagenesis or gene knockout. Extensively used, mutagenesis creates a large and discrete spectrum of lesions that are subsequently detected by forward or reverse genetic approaches. Ethylmethane Sulfonate (EMS) has been a long standing and popular choice to create mutagenized populations in a wide array of species [1–3]. EMS introduces mostly point mutations usually through alkylation of guanine resulting in GC to AT transitions [4]. To date, mostly forward screening approaches have been used in wheat to identify mutants (http://wheat.pw.usda.gov/GG2/Triticum/wgc/2008/). However, owing to the large and complex polyploid genome of wheat, phenotypic screening-based forward genetic approaches are less likely to identify mutants for agronomically relevant genes. Targeting Induced Local Lesions IN Genomes (TILLING), one of the reverse genetic approaches, is often the method of choice to identify mutants in a target gene of known sequence [5–7]. TILLING combines the use of chemical mutagenesis followed by application of mismatch detection assays or high throughput sequencing of PCR amplicons. Along with the requirement of prior gene sequence information to generate PCR primers, TILLING entails significant time and effort and seems to be a prohibitive approach in crops where genome sequence is not available. In bread wheat (*Triticum aestivum* L.), despite the availability of considerable sequence information in Expressed Sequence Tag (EST) database, it is difficult to design genome-specific PCR primers because of the presence of three very closely related homoeologs.

A diverse set of publicly available mutants is needed in wheat to help assign function to its genes including potential candidates of agronomic importance. Recent technical and bioinformatic advancements have made DNA sequencing cheaper and more robust but not enough to perform complete genome sequencing of a large sized mutant population. Thus, a rapid, facile, high throughput, and cost effective method to detect induced mutations in the polyploid genome of wheat is necessary. Contemporary improvements in terms of high throughput sequencing platforms have facilitated the discovery of large-scale natural variation in several genomes including model [8] as well as crop plants such as maize, sorghum, and soybean in a time and cost effective manner [9–11]. Sophisticated algorithms with improved accuracy in SNP calling have been developed and are being applied to identify induced point mutations in diploid and tetraploid species [12, 13]. Applying high throughput sequencing technologies coupled with variant detection software will likely prove to be efficient in detecting mutations in bread wheat as well. Furthermore, sequence-based detection of mutations will bypass the pre-requisites such as genome-specific markers needed for TILLING and other currently used mutation detection methods.

Bread wheat has a very large genome [17 billion basepairs (bp)] of which it is estimated that 1–5 % represents coding sequence [14, 15]. Since mutations present in the coding regions of the genome are of main interest, any sequencing-based mutant detection strategy has to target the coding part of the genome. The use of reduced representation libraries followed by sequencing [Genotyping by Sequencing (GBS)] is one such approach [9]. It reduces the genome complexity and targets the genic part of the genome by the use of methylation sensitive restriction enzymes. This approach also allows sample pooling that further reduces the per sample cost and it has been successfully used to assess natural sequence variation in a number of species including switchgrass [16], soybean [17], maize [9], Arabidopsis, and lettuce [18]. It should be equally beneficial to detect sequence variation present in mutant populations.

With a long-term objective of developing a community resource to quickly identify mutants for a target gene; here we report an efficient approach to identify induced mutations by accurately differentiating those from the sequence changes present among the three wheat homoeologs. The efficiency of the bioinformatics pipeline was tested by detecting the known sequence changes in the three wheat genes with known sequence for all the homoeologs and paralogs.

## Results

### Unigene-based reference sequence for hexaploid wheat

As an ordered genome sequence is not available for hexaploid wheat, we used the NCBI Unigene Build # 63 (http://www.ncbi.nlm.nih.gov/UniGene/UGOrg.cgi?TAXID=4565; as of April 2013) as a reference transcriptome. As unigenes represent only the genic portion, thus the complexity of the wheat genome is further reduced. Dataset contained 1,551,792 sequences of which 35 % were mRNA sequences, 26 % 5′ ESTs, 20 % other ESTs, 18 % 3′ ESTs, and 1 % high-throughput cDNA (HTC) sequences. These sequences encompassed key developmental stages of wheat from cultivars Chinese Spring, Recital, KitaKEI1354, Norstar, Valuevskaya, Thatcher, Atlas66, Scout66, and Stephens. Alignment and clustering by NCBI of these sequences resulted in 178,464 unique contigs, of which about 60 % were singletons and 40 % contained multiple sequences. Functional annotation was available for merely 1.8 % of the unigenes. The unigene length ranged from 103 bp to 11,803 bp with an average length of about 620 bp. About 40 % of the unigenes were longer than 500 bp whereas the length of the remaining 60 % ranged from 103 bp to 500 bp. The unigene set covered about 110 million basepairs (Mbp) of the genic part of the wheat genome.

### Mapping of the sequence reads

**Fig. 1:**
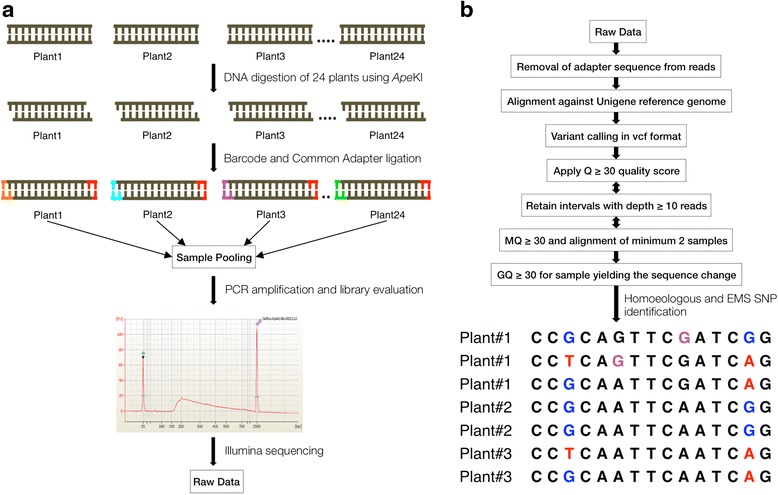
Schematic diagram for mutation detection using novel application of Genotyping-by-sequencing (GBS). **a** DNA of 24 plants including 22 EMS generated M2 and two wild types was digested with *Ape*KI followed by unique barcode (highlighted in *orange*, *cyan*, *purple*, and *green*) and common adapter (highlighted in *red*) ligation. All samples were pooled together before PCR amplification. Pooled sample library was evaluated for its quality and size. Sequencing of the library was performed on Illumina HiSeq2000. **b** Raw data files processed according to the filters described in Methods section. Differentiation of EMS SNPs from homoeologous SNPs is shown from three mutant plants where homoeologous SNP positions are marked in *red* and *blue* while mutational SNPs are highlighted in *purple*

Genotyping-by-sequencing of the 24-plant *Ape*KI library using Illumina HiSeq2000 platform (Fig. 1a) generated a total of 223,301,699 reads. About 97 % of the reads contained the barcode and of these the 74.2 % containing the remnant *Ape*KI cut site were selected (Additional file 1) (see Methods). These 167,389,217 reads were further sorted into 24 files according to their barcode using a custom written Perl script. Plant # 15 had poor quality reads, thus it was not used for further analysis, reducing the dataset to 23 plants. The barcodes were removed and the read sequences were trimmed to 64 bases (including the initial CWGC) (Fig. 1b). The number of reads among mutant plants ranged from 114,538 (plant # 31) to 15,294,951 (plant # 35) whereas the average number of reads for the two wild type plants was 2,443,718 (Additional file 1). Using the Burrows-Wheeler Aligner (BWA) [19], these reads were aligned with the NCBI wheat unigene-based reference sequence (110 Mbp). A very narrow range of mapped reads per plant was observed (7.8–10.5 %) (Additional file 1) and overall 9 % of the filtered reads mapped to the reference unigene sequence. Among the 23 plants, the number of identified unigenes ranged from 1,601 (plant # 31) to 47,880 (plant # 27) consistent with the number of reads generated from these plants (Additional file 1).

Sequencing reads of all mutants and two wild type plants were submitted to the National Center for Biotechnology Information (NCBI) Sequence Read Archive (study SRP056743).

### Sequence coverage and depth of unigenes

**Fig. 2:**
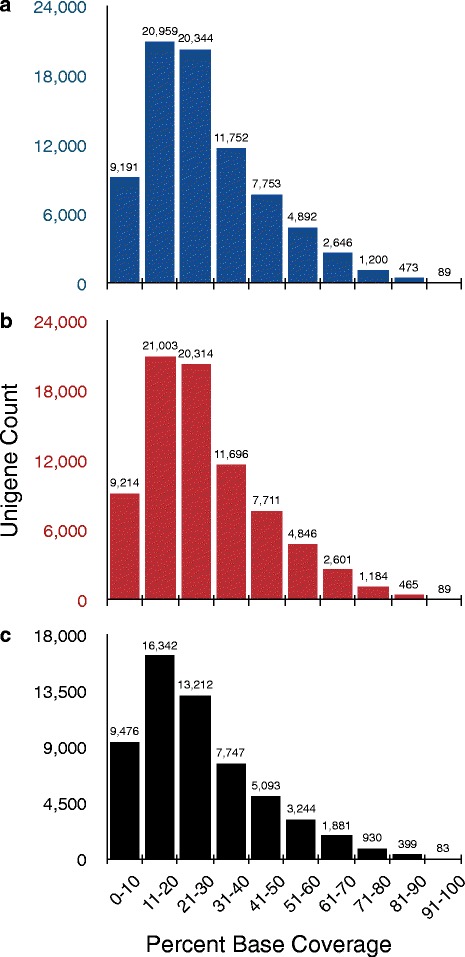
Distribution of mapped unigenes according to different classes of percent base coverage. **a** Mapped unigenes from 21 mutant and 2 wild plants. **b** Mapped unigenes from 21 mutant plants only. **c** Mapped unigenes from 21 mutant plants only when minimum depth was set to 10. X-axis represents the range of classes based on percent base coverage and Y-axis represents the mapped unigene count

Selected reads from the 21 mutant and the two wild type plants aligned with 79,299 unigenes with an average unigene coverage of 28 % (Fig. 2a). Not including the reads from wild type plants, all the mutants reads aligned with 79,123 unigenes with an average coverage of 27 % (Fig. 2b). The unigenes coverage varied from 0 to 100 % with a maximum number of unigenes (20,959 and 21,003 in Fig. 2a and b, respectively) with 11–20 % base coverage. Eighty-eight percent (69,999 and 69,938 in Fig. 2a and b, respectively) of the unigenes had coverage up to 50 % with the mapped reads. A similar distribution of unigenes in different base percentage coverage class is observed for 21 pooled mutants with minimum depth of 10 reads (Fig. 2c).

**Fig. 3:**
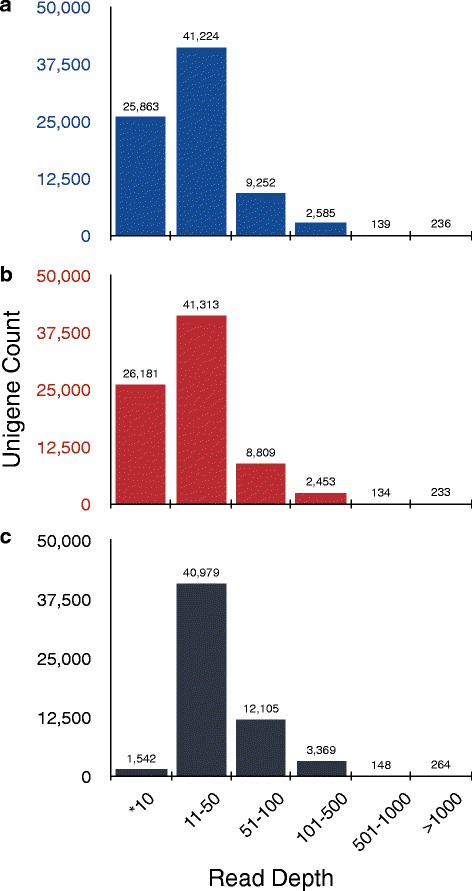
Distribution of mapped unigenes according to different classes of read depth. **a** Mapped unigenes from 21 mutant and 2 wild plants. **b** Mapped unigenes from 21 mutant plants only. **c** Mapped unigenes from 21 mutant plants only when minimum depth was set to 10. X-axis represents the range of classes for read depth and Y-axis represents the mapped unigene count. Asterisk denotes range of one to ten reads in **a** and **b** and only ten reads in **c**

Considering 23 plants, the read depth per unigene ranged from one to >1000. For 33 % (25,863) of the unigenes, the reads depth per unigene was ≤10 whereas for 52 % (41,224) of the unigenes, the number ranged from 11 to 50 (Fig. 3a). A similar pattern of read depth distribution per unigene was observed considering the reads from 21 mutants (Fig. 3b). 74 % (58,407 in number) of the unigenes from 21 mutants were retained after filtering for a minimum depth of 10 reads per unigene and the maximum number of unigenes (40,979) fall into 11–50 read depth class (Fig. 3c).

Reads from the 23 plants were also sequentially aligned to analyze the pattern of change of depth and coverage for the mapped portions of the unigenes. Sequential addition of samples was performed starting with the plant containing the least number of mapped reads to 1,601 unigenes and culminating with the plant containing the highest number of mapped reads to 47,880 unigenes. The number of unique unigenes increased with the increase in each sample addition up to a certain limit. Thereafter, the number of unigenes increased at a decreasing rate. The percent unigene sequence coverage followed the same pattern whereas the depth per unigene showed the opposite pattern (data not shown).

To verify that our pipeline is capturing all the sequence changes including homoeologous as well as mutational, a read depth analysis with constant coverage of 64 bp was performed (Additional file 2). The number of captured changes per read were significantly higher when read depth was increased from 10 to 20, however, the number of changes per read remained same with depth ≥ 20.

### Differentiating mutations from homoeologous changes

Composite alignments of the selected reads from the 23 plants with the unigene set were evaluated based on different parameters in SAMtools (see methods for details).

Alignment of all the selected reads from 23 plants with the unigenes revealed a total of 359,769 variants including 96.74 % SNPs and 3.26 % INDELs. Application of a QUAL ≥ 30 filter reduced the number of variants to 240,439, including 96.48 % SNPs and 3.52 % INDELs. Of these sequence changes, 204,466 met the criteria of DP10 per variant location and MQ ≥ 30. Further, using the criteria of requiring reads from two or more plants in an alignment around a sequence change, 201,353 variants were retained. 150,511 of these changes were sequence differences among wheat homoeologs as were present in more than one plant. About 97.8 % of these changes were SNPs and 2.2 % were INDELs. Additionally, intervarietal changes constituting 33,899 SNPs and 2,813 INDELs were identified. The remaining 14,130 sequence changes present in only one of the plants were thus categorized as EMS mutations (see methods). About 98 % (13,912) of these changes were SNPs and the remaining 2 % (218) were INDELs. Out of 13,912 SNP changes, homozygous mutations represented as 1/1 or 0/0 only in single plant constituted 11 % and heterozygous mutations represented as 0/1 only in single plant constituted 89 %.

**Fig. 4:**
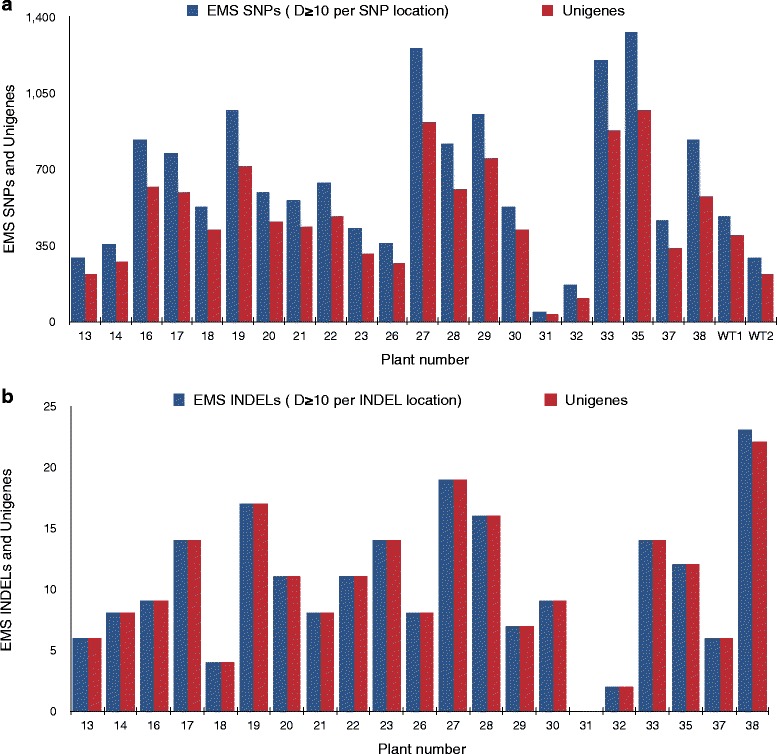
Distribution of EMS induced changes and unigenes in mutant plants at read depth (DP) ≥10 (**a**) EMS induced SNPs and the unigenes harboring these SNPs. **b** EMS induced INDELs and the unigenes harboring these INDELs. Blue bar represents the number of EMS SNPs in **a** and INDELs in **b** Red bar represents the number of unigenes hit in **a** and **b**

Overall, 14,130 mutational changes were observed in 7,027 wheat unigenes with an average of two changes/unigene. For individual plants, the number of unigenes showing mutational changes ranged from 29 in plant # 31 to 980 in plant # 35, consistent with the number of reads generated in these samples. Of these mutational changes, the smallest number of mutational SNPs was observed in plant # 31 with only 46 sequence changes. The highest number of 1,330 SNPs was observed in plant # 35 (Fig. 4a). On an average, 662 induced SNP changes were observed for each plant. The average number of unigenes showing mutational SNPs per plant was 494. Similarly, a total of 218 EMS induced INDELs were identified ranging from 0 (plant # 31) to 23 (plant # 38) with 10 INDELs per plant (Fig. 4b). Of these, 79 of the INDELs were due to insertions and 139 were due to deletions. The size of deletions ranged from one to three bp and the size range for the insertions was one to four bp.

### Characterization of the SNPs induced by EMS

**Fig. 5:**
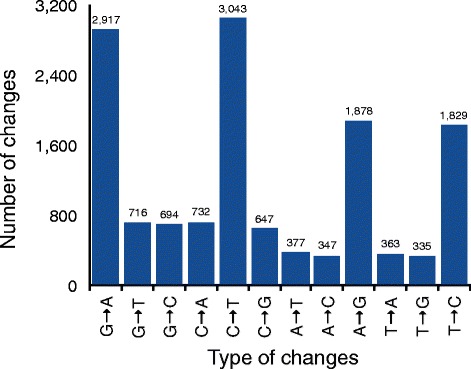
Characterization of SNPs induced by EMS. X-axis denotes the type of changes and Y-axis denotes the number of changes of each type

Among all single base changes induced by EMS, 69.5 % (9,667) were transitions and 30.5 % (4,245) were transversions (Fig. 5). The most frequent transition was C → T (31.5 %) followed by G → A (30.2 %), A → G (19.4 %), and T → C (18.9 %). Among transversions, C → A (17.2 %) was the most frequent followed by G → T (16.9 %), G → C (16.4 %), and C → G (15.2 %). The frequency of other transversions was: A → T (8.9 %), T → A (8.6 %), A → C (8.2 %), and T → G (7.9 %).

For each unigene hit by EMS, the longest stretch of open reading frame was extracted using a custom perl script to categorize these sequence changes as synonymous or non-synonymous. 37 % were found to be synonymous and 63 % as non-synonymous.

### Distribution of EMS induced changes

**Fig. 6:**
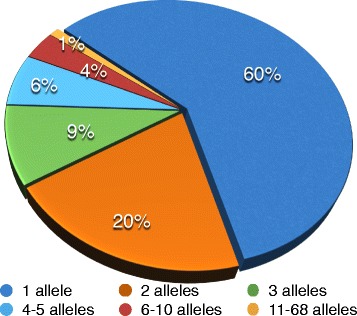
Distribution of allelic variation induced by EMS in unigenes. Proportion of unigenes carrying different number of alleles is shown in different colors

The 13,912 EMS-induced SNPs were present in 6,961 unigenes with an average frequency of two/unigene. The number of EMS-induced SNPs/unigene, referred as alleles, ranged from 1 to 68 (Fig. 6). About 60 % of the 6,961 unigenes had only one SNP change and 20 % had two changes. 15 % of the unigenes had three to five alleles per unigene. Only 5 % of unigenes showed more than six changes per unigene. The 218 EMS induced INDELs were present in 212 unigenes with an average frequency of one/unigene. 146 unigenes had both INDELs and EMS induced SNPs. Further, the unigenes harboring both INDELs and SNPs had an average of six SNPs compared to two in the unigenes containing only SNPs.

Out of 7,027 unigenes with EMS changes, functional annotation was available only for 240 unigenes (wheat unigene build # 63). Therefore, we classified these unigenes based on the annotations given to rice genes using a highly stringent e-value of < 1e-80 using standalone NCBI batch blast (ftp://ftp.ncbi.nlm.nih.gov/blast/executables/LATEST). As a result, an additional 2,332 unigenes were annotated and a large proportion (46 %) of which were classified as genes performing metabolic processes followed by 22 % annotated as expressed proteins. 10 % of each fell under the category of transcription factors and transposable elements (TE). To localize the EMS hit unigenes on the wheat chromosomes, *in-silico* mapping was performed using the mapped wheat EST database [20]. Using consensus physical maps, a total of 5,500 ESTs were mapped on seven groups of chromosomes (Table 1). Standalone batch blast performed using fasta sequences of 6,961 EMS hit unigenes with the mapped 5,500 EST sequences at an e-value of ≤ 1e-100 assigned mapping position to 617 unigenes. Mutations within each chromosome arm region were normalized to the total number of unigenes mapped to that region. Wheat homologous group 2, 3 and 4 are more vulnerable to EMS induced changes compared to group 1 and 5. Homologous group 6 and 7 showed intermediate susceptibility for mutational change (Table 1). For homologous group 1 and 2, more number of mutations was mapped on short arm whereas for chromosome group 4, 5, and 7, more number of mutations was mapped on long arm. For chromosome 3 and 6, mutations were equally distributed on both arms. By and large, genes present in the distal regions of the chromosomes showed a higher tendency to be hit by EMS followed by middle region and the least susceptibility shown by the genes present in the proximal regions (Table 1).

**Table 1:** Physical mapping of EMS induced mutations to three regions on each arm of consensus chromosomes of all 7 groups of wheat

Region	Mapped ESTs	Observed mutations	Normalized mutations	Mutations/arm	Mutations/chromosome
R1 (C-1 L-0.32)	86	0	0.00	0.09	0.61
R2 (1 L-0.32–0.61)	117	2	0.02		
R3 (1 L-0.61–1.00)	203	14	0.07		
R1 (C-1S-0.48)	39	4	0.10	0.52	
R2 (1S-0.48–0.70)	30	9	0.30		
R3 (1S-0.70–1.00)	214	26	0.12		
R1 (C-2 L-0.36)	50	5	0.10	0.59	1.28
R2 (2 L-0.36–0.76)	224	34	0.15		
R3 (2 L-0.76–1.00)	229	77	0.34		
R1 (C-2S–0.33)	50	6	0.12	0.69	
R2 (2S-0.33–0.75)	98	27	0.28		
R3 (2S-0.75–1.00)	161	47	0.29		
R1 (C-3 L-0.27)	106	11	0.10	0.58	1.18
R2 (3 L-0.27–0.63)	125	25	0.20		
R3 (3 L-0.63–1.00)	328	91	0.28		
R1 (C-3S–0.33)	82	10	0.12	0.60	
R2 (3S-0.33–0.78)	156	38	0.24		
R3 (3S-0.78–1.00)	81	19	0.23		
R1 (C-4 L-0.31)	42	14	0.33	0.73	1.12
R2 (4 L-0.31–0.66)	272	49	0.18		
R3 (4 L-0.66–1.00)	360	78	0.22		
R1 (C-4S–0.37)	57	3	0.05	0.39	
R2 (4S-0.37–0.67)	52	10	0.19		
R3 (4S-0.67–1.00)	238	35	0.15		
R1 (C-5 L-0.35)	31	2	0.06	0.38	0.69
R2 (5 L-0.35–0.75)	94	13	0.14		
R3 (5 L-0.75–1.00)	395	70	0.18		
R1 (C-5S-0.40)	21	0	0.00	0.31	
R2 (5S-0.40–0.71)	75	10	0.13		
R3 (5S-0.71–1.00)	113	20	0.18		
R1 (C-6 L-0.36)	55	8	0.15	0.50	1.00
R2 (6 L-0.36–0.68)	69	16	0.23		
R3 (6 L-0.68–1.00)	159	20	0.13		
R1 (C-6S-0.35)	43	8	0.19	0.50	
R2 (6S-0.35–0.76)	70	8	0.11		
R3 (6S-0.76–1.00)	149	30	0.20		
R1 (C-7 L-0.33)	77	19	0.25	0.54	0.98
R2 (7 L-0.33–0.71)	126	18	0.14		
R3 (7 L-0.71–1.00)	212	32	0.15		
R1 (C-7S-0.36)	106	22	0.21	0.44	
R2 (7S-0.36–0.61)	93	14	0.15		
R3 (7S-0.61–1.00)	212	18	0.08		

### Identification of mutations in target genes

We tested accuracy of our pipeline to differentiate mutational changes from homoeologous changes; and evaluated the possibility of identifying mutations in target genes by considering three genes viz. *Rht1* (Reduced height1), *TaABCB1* (ATP binding cassette type B1), and *C-Ph1* (Pairing homoeologous1) [21] for which complete gene sequence was available from the three genomes of hexaploid wheat. The accession numbers for *Rht1* – A, B and D copies are JF930277, JF930278 and HE585643, respectively. The ability of the pipeline to correctly detect homoeologous SNPs was tested on all the intervals of these genes where the sequenced reads were mapped. With a minimum depth of 10 reads, maximum coverage was 65 % for *Rht1* (Additional file 3), followed by 26 % for *TaABCB1* (Additional file 4), and 16 % for *C-Ph1* (Additional file 5). Analysis of the covered part of the three copies for each of the gene sequences from cultivar Chinese Spring revealed 43 homoeologous SNPs *for Rht1,* 18 for *TaABCB1*, and six for *C-Ph1.* With the minimum depth of 10 reads for each change, our analysis pipeline revealed 22 homoeologous SNPs for *Rht1* and 14 for *TaABCB1.* None of the six homoeologous changes for *C-Ph1* were detected by our pipeline. One EMS induced SNP was identified for each of the *Rht1* and *TaABCB1* gene while two EMS induced SNPs were identified for C-*Ph1* gene in the EMS treated plants.

Similar analysis performed with redefined parameter (GQ ≥ 20) identified 24 homoeologous changes for *Rht1*, 14 for *TaABCB1*, and three for *C-Ph1*.The number of EMS induced SNPs increased to two for *TaABCB1* and four for *Rht1.* No mistakes were detected with both the stringent (GQ ≥ 30) and redefined (GQ ≥ 20) criteria to detect and differentiate homoeologous and induced sequence changes, thus validating our pipeline to efficiently differentiate homoeologous SNPs from EMS induced changes.

The empirically developed redefined criteria of detecting changes were then used to identify mutational changes. With the new procedure, a total of 27,770 mutational SNPs were identified compared to the 13,912 that were previously detected. The number of unigenes showing mutational changes increased to 12,812 compared to 6,961. Furthermore, in the redefined criteria, the number of EMS induced SNPs, homoeologous SNPs and INDELs, intervarietal SNPs and INDELs increased to 27,770, 156,740 and 4,953, 36,656 and 4,189, respectively. EMS induced SNPs ranged from 79 in plant # 31 to 2,606 in plant # 27 and the average number of EMS SNPs detected was 1,322 from an average of 967 unigenes hit per plant. Though the largest and smallest insertion and deletion remained the same as that of the previous criteria, redefined criteria resulted in 360 more EMS induced INDELs (578) ranging from no INDELs in plant # 31 to 57 in plant # 27. On an average, each plant had 27 INDELs. If only EMS induced SNPs are considered, the allelic variation generated per unigene by EMS varied from a minimum of one to a maximum of 85. The frequency of unigenes’ class carrying single alleles reduced by 4 %, two and three alleles each increased by 1 %, four–five alleles increased by 1.5 %, six–ten alleles remained same, and 11–85 alleles increased by 0.5 % as compared to the previous classification.

### Mutation rate and EMS induced mutation calling error rate

Alignment of reads from all mutant plants with the wheat unigenes covered 72,558,853 bp. With the first criteria, 14,130 mutational changes were identified, thus, resulting in one mutational change per 5.1 Kb of the wheat genome. With the redefined criteria, 28,348 mutational changes were identified equaling one change per 2.6 Kb. Only 4 % (7,027 unigenes out of total 178,464 unigenes) of the wheat genes showed mutational changes in 21 mutagenized plants. Error in mutation calling was determined by utilizing the stringent criterion in the Variant calling protocol (see Methods) for wild type plants. Using the retained variants of 201,353 after application of QUAL ≥ 30, DP10 per variant location, MQ ≥ 30, and presence of reads from two or more plants in an alignment, the number of variants showing presence of wild-type reads as well were determined. Wild-type reads were present for 57,520 variants. Any mutation showing up in the wild type plants can only be due to error. Treating the wild-type plants in a similar manner to mutant plants, if we found an occurrence of 0/0, 0/1, or 1/1 only in the wild type plant as compared to a uniform genotype for the rest of the mapped samples, we considered it as an error in calling EMS induced mutation. For 777 variants out of 57,520, 0/0, 0/1, or 1/1 genotype was present only in the wild type plant resulting in an error rate 1.35 %. While using the redefined criteria an error rate of 1.45 % was observed in the wild type plant.

## Discussion

TILLING has been a popular method to identify mutations in a target gene sequence. The selected plants undergo extensive characterization and the identified mutants are eventually confirmed by sequencing of the target gene. TILLING requires a significant amount of time to identify mutants for a large number of genes. With plummeting sequencing costs, sequencing based genotyping is routinely used and mutation detection is beginning to rise [13, 22]. Here application of a GBS approach has been tested and shown for the first time to be an efficient method for the discovery of induced mutations in polyploid species such as wheat. The discovery of induced mutations using this method and the analysis pipeline are expected to increase in wheat with the availability of genome sequence for all the genes in wheat. The method described here and the pipeline developed can be easily utilized for diploid species. Large scale mutation detection is feasible using GBS as we have identified nearly five times larger number of genes than that of previous studies [23].

Despite the equal amount of DNA used per plant, the number of reads generated in individual samples composing the *Ape*KI library had a higher coefficient of variation than reported in Elshire et al. 2011. However, a 12-fold difference was observed in the number of reads generated in a barley experiment [24]. Also, different sequencing platforms can result in a large variation in the number of reads generated per sample [25]. One limitation of GBS for mutation detection using single-end reads is that it is not possible to remove duplicate reads. Non-removal of duplicate reads could increase the errors caused by PCR but removal of these reads would also significantly decrease the depth and subsequently loss in identification of low level mutations. Irrespective of the number of reads generated per sample, the number of mapped reads showed a consistent value (9–10 %). However, the number of EMS induced changes increased proportionally in the plants containing higher number of selected reads. Alignment of only 15,065,030 reads to the reference transcriptome can be attributed to the lack of a comprehensive wheat reference genome. In order to investigate how much over or under representation is exhibited by wheat unigenes in the form of 178,464 sequences, batch blast was performed using three types of rice databases viz. rice CDS consisting of 55,986 loci including 16,941 TE loci, rice CDS consisting of only 39,045 non-TE loci and 35,679 cDNA sequences as query against wheat unigenes. Comparison of these datasets was performed under different e-values ranging from ≤ 1e-10 to ≤ 1e-80 (Additional file 6). 60 % of the rice genes were covered with the wheat unigenes. The paradigm shown by adding sequential addition of samples for unique unigenes, unigene sequence coverage, and depth suggests that higher multiplexing can be done during library preparation while cutting the cost of mutation detection to redistribute the number of reads towards coverage rather than depth. However, more coverage of unigenes may also be targeted by using a two enzyme GBS approach [26].

Next-generation sequencing experiments are becoming a routine to find rare or novel variants leading to disease problems in humans [27, 28]. However, sequencing artifacts are naturally associated with these experiments and can conceal the identification of true variants. Usually, two main parameters – read depth and phred score are employed to curtail the variants [29–32]. The high penalty imposed by these filters often removes the real variants. To overcome these limitations, we used these parameters in a more liberal way. However, we added an additional set of filters due to the hexaploid nature of the wheat genome. By using a combination of moderate read depth, phred score, MQ 30 and presence of EMS induced variant only in single plant, we separated a large portion of the homoeologous variants, while retaining the highest possible number of EMS induced variants.

For the variant to be induced by EMS, we computed the GQ for the plant yielding the change which is based on the likelihood of a particular genotype being called in comparison to the likelihood of the other two genotypes being called: L(0/1) versus L(0,0) and L(1,1). The low GQ suggests a low confidence in those calls, which may be due to poor individual sequencing reads. So all the EMS variants were retained that showed a high GQ (30). We chose to have a read depth of 10 assuming at least 3X coverage from each of the three homoeologous copies. EMS induced change was called when the change was present only in single plant as it is very unlikely that a similar mutation will be consistently produced at the same particular location. In addition to rapid identification, sequencing based mutation detection also offers simultaneous mapping of these mutations.

We expect that one of the possibilities of not detecting all the homoeologous SNPs in the three genes tested with the redefined criteria is due to the alignment of only those copies of the gene that do not possess the homoeologous SNP or no homoeologous SNP exists at that location due to the intervarietal differences. However, the reads mapped to respective genes showing homoeologous changes can be easily used to generate SNP based markers for genotyping studies, provided those changes are conserved in different cultivars. While various pipelines are available that can be employed to differentiate between true and false variants [33, 34], we focused on developing an analysis pipeline that is easy to use, adopt and applicable particularly for polyploid species.

Our analysis clearly indicates that genes differ in their susceptibility to EMS as the range was much wider (1–68 and 1–85 in first and redefined criteria, respectively) compared to average number of two SNPs/unigene. It is also evident that EMS mutations are unevenly distributed among the wheat genes and on wheat chromosomes. Only 7,027 unigenes out of 79,123 showed EMS changes. Further, genes present at the distal ends of the chromosomes showed higher rates of mutation compared to those present in the proximal region. Characterization of this mutant population through the screening of several unigenes revealed mutation densities of average one mutation per 5 Kb (Additional file 7). This mutation density is higher than those found by earlier studies based on TILLING [7, 35–37]. The expected array of mutations in an EMS-treated population is primarily GC/AT transition. In wheat, the majority of the identified mutations are GC/AT transitions [7]. In this study, we also found a preponderance of GC to AT transition (43 %), though at a lower level. Large deletions (>100 Kb) and insertions (~500 Kb) were reported in rice [13], but our pipeline does not seem to be capable of detecting huge INDELs however it clearly shows small INDELs caused by EMS mutagenesis. The ability to detect two or more allelic versions caused by EMS in 40 % of the unigenes emphasizes the potential of this strategy to accelerate the pace of wheat functional genomics.

Our first analysis identified a total of 13,912 EMS induced SNPs in the 21 plants with a range of 46 to 1,330. With an error rate of 1.33 % and about 63 % of the mutational changes in the non-synonymous bases, the effective EMS induced SNPs would be 8,648. Since, on average 28 % of the unigene sequence was covered for 79,123 unigenes, extrapolation of these numbers for 100 % unigene coverage will result in 30,885 effective number of mutations. For 178,494 unigenes, 69,675 mutational changes are expected.

## Conclusions

This study represents a simple but accurate pipeline to reliably differentiate the mutational changes from the homoeologous changes in a polyploid species. We conclude that sequencing based mutation detection is a valuable method to identify induced mutations at large. By utilizing this reverse genetic approach in the future, more mutagenized populations can be characterized in a time efficient manner. EMS mutations show bias for different genes present at different locations on the chromosome. Genes present at the distal ends of the chromosomes showed higher rate of mutation compared to those present in the proximal regions. Therefore, EMS will serve as a desirable mutagen if the genes of interest are localized towards the telomeric regions.

## Methods

### Plant material and library preparation for sequencing

Twenty-two EMS mutagenized M_2_ plants in the background of hexaploid wheat cultivar ‘Indian’ along with two non-mutagenized control ‘Indian’ plants were used for the study. Genomic DNA was extracted from 2 to 4 week old leaves of the 24 plants, as described earlier [38]. DNA of each plant was normalized to a concentration of 10 ng/μl.

The standard procedure was followed to generate a Genotyping-by-sequencing (GBS) library [9]. Briefly, 24 unique barcoded adapters with 4 to 8 bp barcode (Additional file 8) was generated by mixing of complementary oligonucleotides (Sigma) such that each adapter has an *Ape*KI overhang sequence at its 5′ end. Similarly, the common adapter was generated with *Ape*KI overhang at 5′ end. PCR will amplify only those restriction digested DNA fragments that have barcode adapters added on one end and common adapters added on the other end. The complementary oligonucleotides for each adapter were annealed to a concentration of 300 ng/ul. The annealing was performed by heating the sample to 95 °C for 2 min followed by cooling to 25 °C at the rate of 0.1 °C/s before holding the reaction at 25 °C for 30 min. Both the barcoded and the common adapters were mixed in an equimolar ratio. About 3.6 ng of the mixed adapters was transferred to each of the 24-wells of a 96-well plate and dried by placing the plate in a refrigerator for 2–5 days. 100 ng of genomic DNA for each plant was added to the same 24-wells, dried and was restriction digested at 75 °C for 2 h in 20 μl reactions each containing 1X NEB buffer 3, and 4 U *Ape*KI. In the same wells, adapter ligation was performed by adding 1X T4 DNA ligase buffer, 640 U of T4 ligase in a total volume of 50 μl made with sterile distilled water. After ligation the 24 plants were pooled, purified with QIAquick column (Qiagen PCR cleanup kit) and PCR amplified using the following conditions - 72 °C for 5 min, 98 °C for 30 sec followed by 18 cycles of 98 °C for 30 sec, 65 °C for 30 sec, 72 °C for 30 sec with a final extension at 72 °C for 5 min. Quality of the library was evaluated on Agilent 2100 bioanalyzer (Additional file 9) as described earlier [9].

### DNA sequencing and data processing

Paired-end sequencing (100 bp reads) was performed on a single flowcell channel of HiSeq2000 (Illumina, Inc., San Diego, CA). Initial quality checks were performed using FastQC (http://www.bioinformatics.babraham.ac.uk/projects/fastqc). The single-end barcode sequence data was filtered and trimmed to 64 bp [9]. Only the reads perfectly matching to one of the barcodes and containing the expected four-base remnant of the *Ape*KI cut site (CWGC) were selected. Reads containing “N’s” in the first 72 bases and adapter/adapter dimers were discarded.

### Read alignments to the wheat unigenes

The wheat unigene set consisting of 178,464 sequences was downloaded from NCBI FTP server (ftp://ftp.ncbi.nih.gov/repository/UniGene/Triticum_aestivum/; as of April 2013) and used as a reference transcriptome. Using Burrows–Wheeler string transformation (BWT), the sequence was indexed to accelerate its alignment by transforming the highly redundant sequences into a compressed format. Following the indexing of the reference transcriptome, the reads were then mapped to the reference transcriptome using Burrows-Wheeler alignment tool (BWA 0.5.9) with the default settings and the read alignment data was saved in the SAM (Sequence alignment/map) format. SAMtools ‘flagstat’ command was used to fetch mapping statistics from the binary version i.e. BAM format of the aforementioned format.

### Average coverage and depth of unigenes

Alignment data was processed using SAMtools. This included converting, sorting, and merging the alignment data. The merged sorted.bam file containing mapped reads from the 23 plants and ‘genomecov’ function in BEDTools (version 2.17.0) [39] was used to compute summaries of the aligned sequences coverage for the reference transcriptome. The average depth and coverage per unigene data was computed using custom Perl scripts. For each read-mapping interval, alignment of all three homoeologous copies was tried to achieve by keeping a minimum of 10 reads. Since short read sequencing technology was employed, one to several intervals within a gene were covered by the reads showing end to end as well as overlapping alignment. The percent coverage per unigene was determined by dividing the number of bases covered by the mapped reads (including both the end to end and overlapping alignments) to the total number of bases of the respective unigenes. For depth, reads within the overlapping and non-overlapping alignment regions were counted separately. Then, average depth per unigene was calculated by dividing the number of reads present in all the regions to the number of regions. Further, to calculate the number of unigenes with alignment of 10 or more reads, all the regions within a unigene having less than 10 reads were excluded from the analysis.

### Differentiation between homoeologous and EMS induced changes

Sequence variant detection, genotype calling and EMS induced variant identification were processed with SAMTools (version 0.1.19) [40] ‘*mpileup*’ function and custom Perl scripts. The output of variants was stored in the variant call format (vcf) that consisted of meta-information for individual variant with respect to reference sequence in the form of various parameters. These parameters were represented in different columns in the vcf file as position of the variant in the unigene, reference (REF) allele, alternate (ALT) allele, phred scaled quality score (QUAL), combined depth across samples (DP), number of samples with data (NS), and other relevant information consisting of mapping quality (MQ), genotype (GT) represented by 0 for the REF allele and 1 for the ALT allele, and genotype quality (GQ). 0/0 genotype represents presence of reference alleles only whereas 0/1 represents presence of both wild type as well as alternate allele in the sample, and 1/1 represents presence of only alternate allele in the sample. A variant (SNP/INDEL) was called when occurrence of 0/1 or 1/1 genotype in the mutant plants at a particular position along the length of the unigene was observed as compared to 0/0 genotype for the reference. To avoid/minimize the detection of false positives, only the sequence changes with QUAL ≥ 30 were selected. Furthermore, we required that the aligned reads should have MQ ≥ 30 and at least 10 reads should be aligned at that locus from more than one sample. A change that was present in only one of the mutant plants as compared to a uniform genotype for the rest of the mapped samples was considered to be EMS induced after application of a threshold of GQ ≥ 30. GQ was encoded as a phred quality score (−10log_10_^(*p*)^). A sequence change present in reads corresponding to more than one of the 23 plants was scored as a homoeologous change. In comparison to the unigenes, any sequence change present in all of the reads was categorized as intervarietal changes.

In-house perl scripts developed for this analysis pipeline are available upon request.

#### Mapping of ESTs on consensus physical map

For each of the seven groups of chromosomes, a consensus physical map was constructed. Deletion breakpoints were placed on each of the chromosome arm so as to physically divide it into approximately three equal regions-R1, R2, and R3, where R1 was represented as proximal region to centromere and R3 as distal region. After combining the mapping data from three homoeologs of each chromosome, each EST was localized to the shortest possible chromosome interval and placed on either of the three regions of chromosome arm. ESTs showing discrepancies for location among three homoeologous chromosomes were not considered for analysis.

### Availability of supporting data

The data set(s) supporting the results of this article are included within the additional files of this article.

## Additional files

Additional file 1:
**Is a table listing** **plant-wise** **distribution of reads and their proportion mapped to the respective unigenes.** (PDF 29 kb) Additional file 2:
**Is a figure showing the number of changes captured with constant coverage at varying depth levels.** (PDF 39 kb) Additional file 3:
**Figure showing sequence alignment of three copies of** ***Rht1*** **gene.** (PDF 55 kb) Additional file 4:
**Is a figure showing sequence alignment of three copies of** ***TaABCB1*** **gene.** (PDF 71 kb) Additional file 5:
**Is a figure showing sequence alignment of three copies of** ***C-Ph1*** **gene.** (PDF 45 kb) Additional file 6:
**Is a table listing rice coding sequences (including and excluding TE) and cDNA with wheat unigenes at different** **e-values.** (PDF 35 kb) Additional file 7:
**Is a table showing mutation rate for different mutant plants.** (PDF 33 kb) Additional file 8:
**Is a table showing top and bottom strand barcode adapter sequences with barcode sequence in bold letters.** (PDF 34 kb) Additional file 9:
**Is a figure showing quality check for** ***Ape*** **KI enzyme cut genomic DNA library of hexaploid wheat on a DNA analyzer.** (PDF 71 kb)

## Abbreviations

EMS: Ethylmethane sulfonate
SNPs: Single nucleotide polymorphisms
INDELs: Insertions/deletions
TILLING: Targeting induced local lesions IN genomes
EST: Expressed sequence tag
GBS: Genotyping-by-sequencing
BWA: Burrows-wheeler aligner
MQ: Mapping quality
DP: Depth
GQ: Genotype quality
TE: Transposable elements
*Rht1*: Reduced height1
*ABCB1*: ATP binding cassette type B1
*C-Ph1*: Pairing homoeologous1

## References

[1] Minocha JL, Arnason TJ (1962). Mutagenic effectiveness of ethyl methanesulfonate and methyl methanesulfonate in barley. Nature.

[2] Neuffer MG, Ficsor G (1963). Mutagenic action of ethyl methanesulfonate in maize. Science.

[3] Siddiq EA, Puri RP, Singh VP (1968). Studies on growth and mutation frequency in rice in treatments with dimethyl sulphoxide and ethyl methane sulphonate. Curr Sci.

[4] Krieg DR (1963). Ethyl methanesulfonate-induced reversion of bacteriophage T4rII mutants. Genetics.

[5] McCallum CM, Comai L, Greene EA, Henikoff S (2000). Targeted screening for induced mutations. Nat Biotechnol.

[6] Caldwell DG, McCallum N, Shaw P, Muehlbauer GJ, Marshall DF, Waugh R (2004). A structured mutant population for forward and reverse genetics in barley (*Hordeum vulgare* L.). Plant J.

[7] Slade AJ, Fuerstenberg SI, Loeffler D, Steine MN, Facciotti D (2005). A reverse genetic, nontransgenic approach to wheat crop improvement by TILLING. Nat Biotechnol.

[8] Xu X, Liu X, Ge S, Jensen JD, Hu F, Li X (2012). Resequencing 50 accessions of cultivated and wild rice yields markers for identifying agronomically important genes. Nat Biotechnol.

[9] Elshire RJ, Glaubitz JC, Sun Q, Poland JA, Kawamoto K, Buckler ES (2011). A robust, simple genotyping-by-sequencing (GBS) approach for high diversity species. PLoS One.

[10] Morris GP, Ramu P, Deshpande SP, Hash CT, Shah T, Upadhyaya HD, et al. Population genomic and genome-wide association studies of agroclimatic traits in sorghum. Proc Natl Acad Sci USA. 2013;110:453–8. 10.1073/pnas.1215985110 PMC3545811 23267105

[11] Vidal RO, do Nascimento LC, Mondego JMC, Pereira GAG, Carazzolle MF (2012). Identification of SNPs in RNA-seq data of two cultivars of *Glycine max* (soybean) differing in drought resistance. Genet Mol Biol.

[12] Monson-Miller J, Sanchez-Mendez DC, Fass J, Henry IM, Tai TH, Comai L (2012). Reference genome-independent assessment of mutation density using restriction enzyme-phased sequencing. BMC Genomics.

[13] Henry IM, Nagalakshmi U, Lieberman MC, Ngo KJ, Krasileva KV, Vasquez-Gross H (2014). Efficient genome-wide detection and cataloging of EMS-induced mutations using exome capture and next-generation sequencing. Plant Cell.

[14] Erayman M, Sandhu D, Sidhu D, Dilbirligi M, Baenziger PS, Gill KS (2004). Demarcating the gene-rich regions of the wheat genome. Nucleic Acids Res.

[15] Mayer KFX, Rogers J, Dole El J, Pozniak C, Eversole K, Feuillet C (2014). A chromosome-based draft sequence of the hexaploid bread wheat (*Triticum aestivum*) genome. Science.

[16] Lu F, Lipka AE, Glaubitz J, Elshire R, Cherney JH, Casler MD (2013). Switchgrass genomic diversity, ploidy, and evolution: novel insights from a network-based SNP discovery protocol. PLoS Genet.

[17] Sonah H, Bastien M, Iquira E, Tardivel A, Légaré G, Boyle B (2013). An improved genotyping by sequencing (GBS) approach offering increased versatility and efficiency of SNP discovery and genotyping. PLoS One.

[18] Truong HT, Ramos AM, Yalcin F, de Ruiter M, van der Poel HJA, Huvenaars KHJ (2012). Sequence-based genotyping for marker discovery and co-dominant scoring in germplasm and populations. PLoS One.

[19] Li H, Durbin R (2009). Fast and accurate short read alignment with Burrows-Wheeler transform. Bioinformatics.

[20] Qi LL, Echalier B, Chao S, Lazo GR, Butler GE, Anderson OD (2004). A chromosome bin map of 16,000 expressed sequence tag loci and distribution of genes among the three genomes of polyploid wheat. Genetics.

[21] Bhullar R, Nagarajan R, Bennypaul H, Sidhu GK, Sidhu G, Rustgi S, et al. Silencing of a metaphase I-specific gene results in a phenotype similar to that of the Pairing homeologous 1 (Ph1) gene mutations. 2014;111:14187–92. 10.1073/pnas.1416241111 PMC4191769 25232038

[22] Tsai H, Howell T, Nitcher R, Missirian V, Watson B, Ngo KJ (2011). Discovery of rare mutations in populations: TILLING by sequencing. Plant Physiol.

[23] Greene EA, Codomo CA, Taylor NE, Henikoff JG, Till BJ, Reynolds SH (2003). Spectrum of chemically induced mutations from a large-scale reverse-genetic screen in Arabidopsis. Genetics.

[24] Liu H, Bayer M, Druka A, Russell JR, Hackett CA, Poland J (2014). An evaluation of genotyping by sequencing (GBS) to map the Breviaristatum-e (ari-e) locus in cultivated barley. BMC Genomics.

[25] Mascher M, Wu S, Amand PS, Stein N, Poland J (2013). Application of genotyping-by-sequencing on semiconductor sequencing platforms: a comparison of genetic and reference-based marker ordering in barley. PLoS One.

[26] Poland JA, Brown PJ, Sorrells ME, Jannink J-L (2012). Development of high-density genetic maps for barley and wheat using a novel two-enzyme genotyping-by-sequencing approach. PLoS One.

[27] MacArthur DG, Manolio TA, Dimmock DP, Rehm HL, Shendure J, Abecasis GR (2014). Guidelines for investigating causality of sequence variants in human disease. Nature.

[28] Ezewudo M, Zwick ME (2013). Evaluating rare variants in complex disorders using next-generation sequencing. Curr Psychiatry Rep.

[29] Girard SL, Gauthier J, Noreau A, Xiong L, Zhou S, Jouan L (2011). Increased exonic de novo mutation rate in individuals with schizophrenia. Nat Genet.

[30] Xu B, Roos JL, Dexheimer P, Boone B, Plummer B, Levy S (2011). Exome sequencing supports a de novo mutational paradigm for schizophrenia. Nat Genet.

[31] O’Roak BJ, Vives L, Fu W, Egertson JD, Stanaway IB, Phelps IG (2012). Multiplex targeted sequencing identifies recurrently mutated genes in autism spectrum disorders. Science.

[32] Sanders SJ, Murtha MT, Gupta AR, Murdoch JD, Raubeson MJ, Willsey AJ (2012). De novo mutations revealed by whole-exome sequencing are strongly associated with autism. Nature.

[33] DePristo MA, Banks E, Poplin R, Garimella KV, Maguire JR, Hartl C (2011). A framework for variation discovery and genotyping using next-generation DNA sequencing data. Nat Genet.

[34] Li H (2011). A statistical framework for SNP calling, mutation discovery, association mapping and population genetical parameter estimation from sequencing data. Bioinformatics.

[35] Uauy C, Paraiso F, Colasuonno P, Tran RK, Tsai H, Berardi S (2009). A modified TILLING approach to detect induced mutations in tetraploid and hexaploid wheat. BMC Plant Biol.

[36] Dong C, Vincent K, Sharp P (2009). Simultaneous mutation detection of three homoeologous genes in wheat by High Resolution Melting analysis and Mutation Surveyor. BMC Plant Biol.

[37] Rawat N, Sehgal SK, Joshi A, Rothe N, Wilson DL, McGraw N (2012). A diploid wheat TILLING resource for wheat functional genomics. BMC Plant Biol.

[38] Randhawa HS, Mutti JS, Kidwell K, Morris CF, Chen X, Gill KS (2009). Rapid and targeted introgression of genes into popular wheat cultivars using marker-assisted background selection. PLoS One.

[39] Quinlan AR, Hall IM (2010). BEDTools: a flexible suite of utilities for comparing genomic features. Bioinformatics.

[40] Li H, Handsaker B, Wysoker A, Fennell T, Ruan J, Homer N (2009). The sequence alignment/map format and SAMtools. Bioinformatics.

