# Supplementary material for: Sequencing-based high throughput mutation detection in bread wheat
Source: BMC Genomics. 2015 Nov 17;16:962. doi: 10.1186/s12864-015-2112-1 (PMC4650848; doi:10.1186/s12864-015-2112-1)
Supplement: Additional file 1: — Is a table listing plant-wise distribution of reads and their proportion mapped to the respective unigenes. (PDF 29 kb) [file 12864_2015_2112_MOESM1_ESM.pdf]

Additional data file 1 Plant-wise distribution of reads and their proportion mapped to the respective unigenes

Plant ID	Total Reads	Filtered Reads (% of Total Reads)	Mapped reads (%)	Covered Unigenes (% of Total Unigenes)
13	3,313,202	2,588,174 (78.1)	9.57	17,785 (10.0)
14	5,363,611	4,079,960 (76.1)	8.98	23,037 (12.9)
15	3,826,793	2,934,236 (76.7)	8.96	20,540 (11.5)
16	11,966,118	9,210,400 (77.0)	9.24	38,155 (21.4)
17	13,125,206	10,219,522 (77.9)	8.72	39,532 (22.2)
18	8,056,055	6,382,143 (79.2)	9.22	29,777 (16.7)
19	14,509,765	11,361,066 (78.3)	9.46	42,410 (23.8)
20	9,753,393	7,619,912 (78.1)	8.51	30,935 (17.3)
21	8,003,163	6,105,060 (76.3)	9.57	31,796 (17.8)
22	9,552,291	7,304,000 (76.5)	8.85	33,034 (18.5)
23	4,665,126	3,677,982 (78.8)	9.21	21,672 (12.1)
26	5,382,041	4,219,632 (78.4)	8.58	24,743 (13.9)
27	19,216,178	15,037,955 (78.3)	8.78	47,880 (26.8)
28	12,925,032	10,044,347 (77.7)	9.03	39,862 (22.3)
29	16,718,597	12,986,973 (77.7)	8.80	41,863 (23.5)
30	8,853,630	6,798,781 (76.8)	9.07	31,717 (17.8)
31	155,345	114,538 (73.7)	9.97	1,601 (0.9)
32	889,931	669,441 (75.2)	9.41	6,243 (3.5)
33	15,028,705	11,267,849 (75.0)	10.46	46,707 (26.2)
35	20,312,263	15,294,951 (75.3)	8.87	47,691 (26.7)
37	5,900,892	4,615,053 (78.2)	8.42	23,376 (13.1)
38	12,710,681	9,969,807 (78.4)	7.81	33,832 (19.0)
Indian	4,511,782	3,471,080 (76.9)	8.87	20,592 (11.5)
Indian	1,852,855	1,416,355 (76.4)	8.89	10,865 (6.1)
Unassigned	6,709,044	49,203,438		
