# Supplementary material for: Sequencing-based high throughput mutation detection in bread wheat
Source: BMC Genomics. 2015 Nov 17;16:962. doi: 10.1186/s12864-015-2112-1 (PMC4650848; doi:10.1186/s12864-015-2112-1)
Supplement: Additional file 2: — Is a figure showing the number of changes captured with constant coverage at varying depth levels. (PDF 39 kb) [file 12864_2015_2112_MOESM2_ESM.pdf]

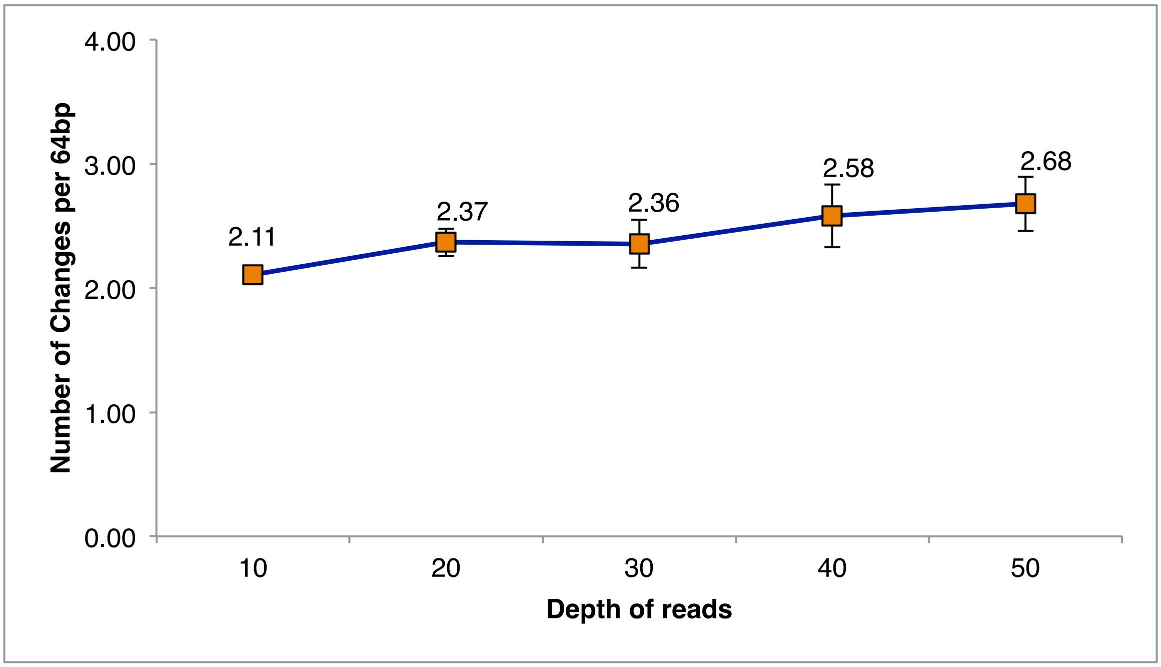

Additional data file 2 The number of changes captured with constant coverage at varying depth levels. Y-axis represents average number of changes and X-axis represents different read depths.
