# Supplementary material for: Sequencing-based high throughput mutation detection in bread wheat
Source: BMC Genomics. 2015 Nov 17;16:962. doi: 10.1186/s12864-015-2112-1 (PMC4650848; doi:10.1186/s12864-015-2112-1)
Supplement: Additional file 3: — Figure showing sequence alignment of three copies of Rht1 gene. (PDF 55 kb) [file 12864_2015_2112_MOESM3_ESM.pdf]

```
Rht1-A   ATGAAGCGCGAGTACCAGGACGCCGGCGGGAGCGGTGGCGGGGG---CGGCATGGGCTCG 57
Rht1-B   ATGAAGCGCGAGTACCAGGACGCCGGAGGGAGCGGCGGTGGCGGGGGAGGCATGGGCTCG 60
Rht1-D   ATGAAGCGGGAGTACCAGGACGCCGGAGGGAGCGGCGGCGGCGGTGGCGGCATGGGCTCG 60
         ******** ***************** ******** ** ** **    ************

Rht1-A   TCTGAGGACAAGATGATGGTGTCGG------CGGCGGCGGGGGAGGGGGAGGAGGTGGAC 111
Rht1-B   TCCGAGGACAAGATGATGGTGTCGGGGTCGGCGGCGGCGGGGGAGGGGGAGGAGGTGGAC 120
Rht1-D   TCCGAGGACAAGATGATGGTGTCGG------CGGCGGCGGGGGAGGGGGAGGAGGTGGAC 114
         ** **********************      *****************************

Rht1-A   GAGCTGCTGGCGGCGCTCGGGTACAAGGTGCGGGCCTCCGACATGGCGGACGTGGCGCAG 171
Rht1-B   GAGCTGCTGGCGGCGCTCGGGTACAAGGTGCGGGCGTCCGACATGGCGGACGTGGCGCAG 180
Rht1-D   GAGCTGCTGGCGGCGCTCGGGTACAAGGTGCGCGCCTCCGACATGGCGGACGTGGCGCAG 174
         ******************************** ** ************************

Rht1-A   AAGCTGGAGCAGCTGGAGATGGCCATGGGGATGGGCGGCGTGGGCGCCGGCGCCGCCCCC 231
Rht1-B   AAGCTGGAGCAGCTGGAGATGGCCATGGGGATGGGCGGCGTGGGCGCCGGCGCCGCGCCC 240
Rht1-D   AAGCTGGAGCAGCTCGAGATGGCCATGGGGATGGGCGGCGTGGGCGCCGGCGCCGCCCCC 234
         ************** ***************************************** ***

Rht1-A   GACGACAGCTTCGCCACCCACCTCGCCACGGACACCGTGCACTACAACCCCACCGACCTC 291
Rht1-B   GACGACAGCTTCGCCACCCACCTCGCCACGGACACCGTGCACTACAACCCCACCGACCTC 300
Rht1-D   GACGACAGCTTCGCCACCCACCTCGCCACGGACACCGTGCACTACAACCCCACCGACCTG 294
         ***********************************************************

Rht1-A   TCCTCCTGGGTCGAGAGCATGCTGTCGGAGCTCAACGCGCCGCCGCCGCCCCTCCCGCCC 351
Rht1-B   TCCTCCTGGGTCGAGAGCATGCTGTCGGAGCTCAACGCGCCGCCGCCGCCCCTCCCGCCC 360
Rht1-D   TCGTCTTGGGTCGAGAGCATGCTGTCGGAGCTCAACGCGCCGCCGCCGCCCCTCCCGCCC 354
         ** ** ******************************************************

Rht1-A   GCCCCGCAGCAGCTCAACGCCTCCACCTCCTCCACCGTCACGGGC---GGTGGGTACTTC 408
Rht1-B   GCCCCGCA---GCTCAACGCCTCCACCTCCTCCACCGTCACCGGC---GGCGGGTACTTC 414
Rht1-D   GCCCCGCA---GCTCAACGCCTCCACCTCCTCCACCGTCACGGGCAGCGGCGGCTACTTC 411
         ********   ****************************** ***   ** ** ******

Rht1-A   GATCTCCCGCCCTCGGTTGACTCCTCCTGCAGCACCTACGCCCTGCGGCCGATCCCCTCC 468
Rht1-B   GATCTCCCGCCCTCCGTCGACTCCTCCTGCAGCACCTACGCGCTGCGGCCGATCCCGTCC 474
Rht1-D   GATCTCCCGCCCTCCGTCGACTCCTCCAGCAGCATCTACGCGCTGCGGCCGATCCCCTCC 471
         ************** ** ********* ****** ****** ************** ***

Rht1-A   CCGGCCGGCGCCGTCGGGCCGGCCGACCTGTCCGCCGACTCCGT---GCGGGACCCCAAG 525
Rht1-B   CCGGCCGTCGC------GCCGGCCGACCTCTCCGCCGACTCCGTCGTGCGGGATCCCAAG 528
Rht1-D   CCGGCCGGCGCGACGGCGCCGGCCGACCTGTCCGCCGACTCCGT---GCGGGATCCCAAG 528
         ******* ***      ************ **************   ****** ******

Rht1-A   CGGATGCGCACTGGCGGGAGCAGCACCTCGTCGTCGTCATCCTCCTCGTCCTCTCTCGG- 584
Rht1-B   CGGATGCGCACTGGCGGCAGCAGCACCTCGTCGTCA---TCCTCATCGTCCTCTCTCGGC 585
Rht1-D   CGGATGCGCACTGGCGGGAGCAGCACCTCGTCGTCATCCTCCTCCTCGTCGTCTCTCGG- 587
         ***************** *****************    ***** ***** ********

Rht1-A   --TGGGGGCGCCAGGAGCTCTGTGGTGGAGGCTGCTCCGCCGGTCGCGGCCGGGGCCAAC 642
Rht1-B   GGTGGCGGCGCCAGGAGCTCTGTGGTGGAGGCTGCCCCGCCGGTGGCCGCCGCGGCCGGT 645
Rht1-D   --TGGGGGCGCCAGGAGCTCTGTGGTGGAGGCTGCCCCGCCGGTCGCGGCCGCGGCCAAC 645
           *** ***************************** ******** ** **** ****

Rht1-A   GCG---CCCGCGCTGCCGGTCGTCGTGGTCGACACGCAGGAGGCCGGGATTCGGCTGGTG 699
Rht1-B   GCG---CCCGCGCTGCCGGTCGTCGTGGTCGACACGCAGGAGGCCGGGATTCGGCTGGTG 702
Rht1-D   GCGACGCCCGCGCTGCCGGTCGTCGTGGTCGACACGCAGGAGGCCGGGATTCGGCTGGTG 705
         ***   ******************************************************

Rht1-A   CACGCGCTGCTGGCCTGCGCGGAGGCCGTGCAGCAGGAGAACTTCTCTGCCGCGGAGGCG 759
Rht1-B   CACGCGCTGCTGGCGTGCGCAGAGGCCGTGCAGCAGGAGAACTTCTCTGCCGCGGAGGCG 762
Rht1-D   CACGCGCTGCTGGCGTGCGCGGAGGCCGTGCAGCAGGAGAACCTCTCCGCCGCGGAGGCG 765
         ************** ***** ********************* **** ************

Rht1-A   CTGGTGAAGCAGATACCCTTGCTGGCCGCGTCCCAGGGCGGCGCGATGCGCAAGGTCGCC 819
Rht1-B   CTGGTGAAGCAGATACCCTTGCTGGCCGCGTCCCAGGGCGGCGCCATGCGCAAGGTCGCC 822
Rht1-D   CTGGTGAAGCAGATACCCTTGCTGGCCGCGTCCCAGGGCGGCGCGATGCGCAAGGTCGCC 825
         ******************************************** ***************

Rht1-A   GCCTACTTCGGCGAGGCCCTCGCCCGCCGCGTCTTCCGCTTCCGCCCGCAGCCGGACAGC 879
Rht1-B   GCCTACTTCGGCGAGGCCCTCGCCCGCCGCGTCTTCCGCTTCCGCCCGCAGCCGGACAGC 882
Rht1-D   GCCTACTTCGGCGAGGCCCTCGCCCGCCGCGTCTTCCGCTTCCGCCCGCAGCCGGACAGC 885
         ************************************************************

Rht1-A   TCCCTCCTCGACGCCGCCTTCGCCGACCTCCTCCACGCGCACTTCTACGAGTCCTGCCCC 939
Rht1-B   TCCCTCCTCGACGCCGCCTTCGCCGACCTCCTCCACGCGCACTTCTACGAGTCCTGCCCC 942
Rht1-D   TCCCTCCTCGACGCCGCCTTCGCCGACCTCCTCCACGCGCACTTCTACGAGTCCTGCCCC 945
         ************************************************************

Rht1-A   TACCTCAAGTTCGCCCACTTCACCGCCAACCAGGCCATCCTGGAGGCCTTCGCCGGCTGC 999
Rht1-B   TACCTCAAGTTCGCCCACTTCACCGCCAACCAGGCCATCCTGGAGGCGTTCGCCGGCTGC 1002
Rht1-D   TACCTCAAGTTCGCGCACTTCACCGCCAACCAGGCCATCCTGGAGGCGTTCGCCGGCTGC 1005
         ************** ******************************** ************
```

```
Rht1-A    CGCCGCGTGCACGTCGTCGACTTCGGCATCAAGCAGGGGATGCAGTGGCCCGCCCTCCTC 1059
Rht1-B    CGCCGCGTGCACGTCGTCGACTTCGGCATCAAGCAGGGGATGCAGTGGCCCGCCCTTCTC 1062
Rht1-D    CGCCGCGTGCACGTCGTCGACTTCGGCATCAAGCAGGGGATGCAGTGGCCCGCACTTCTC 1065
          ***************************************************** ** ***

Rht1-A    CAGGCCCTCGCGCTCCGTCCCGGCGGCCCTCCCTCGTTCCGCCTCACCGGCGTCGGCCCC 1119
Rht1-B    CAGGCCCTGGCGCTCCGTCCCGGCGGCCCTCCCTCGTTCCGCCTCACCGGCGTCGGCCCC 1122
Rht1-D    CAGGCCCTCGCCCTCCGTCCCGGCGGCCCTCCCTCGTTCCGCCTCACCGGCGTCGGCCCC 1125
          ******** ** ************************************************

Rht1-A    CCGCAGCCGGACGAGACCGACGCCTTGCAGCAGGTGGGCTGGAAGCTCGCCCAGTTCGCG 1179
Rht1-B    CCGCAGCCGGACGAGACCGACGCCTTGCAGCAGGTGGGCTGGAAGCTCGCCCAGTTCGCG 1182
Rht1-D    CCGCAGCCGGACGAGACCGACGCCCTGCAGCAGGTGGGCTGGAAGCTCGCCCAGTTCGCG 1185
          ************************ ***********************************

Rht1-A    CACACCATCCGCGTCGACTTCCAGTACCGCGGCCTCGTCGCCGCCACGCTCGCGGACCTG 1239
Rht1-B    CACACCATCCGCGTCGACTTCCAGTACCGCGGCCTCGTCGCCGCCACGCTCGCGGACCTG 1242
Rht1-D    CACACCATCCGCGTCGACTTCCAGTACCGCGGCCTCGTCGCCGCCACGCTCGCGGACCTG 1245
          ************************************************************

Rht1-A    GAGCCATTCATGCTGCAGCCGGAGGGCGAGGAGGACCCGAACGAGGAGCCCGAGGTAATC 1299
Rht1-B    GAGCCGTTCATGCTGCAGCCGGAGGGCGAGGAGGACCCGAACGAGGAGCCCGAGGTAATC 1302
Rht1-D    GAGCCGTTCATGCTGCAGCCGGAGGGCGAGGAGGACCCGAACGAGGAGCCCGAGGTAATC 1305
          ***** ******************************************************

Rht1-A    GCCGTCAACTCGGTCTTCGAGATGCACCGGCTGCTCGCGCAGCCCGGCGCCCTGGAGAAG 1359
Rht1-B    GCCGTCAACTCGGTCTTCGAGATGCACCGGCTGCTCGCGCAGCCCGGCGCCCTGGAGAAG 1362
Rht1-D    GCCGTCAACTCAGTCTTCGAGATGCACCGGCTGCTCGCGCAGCCCGGCGCCCTGGAGAAG 1365
          *********** ************************************************

Rht1-A    GTCCTGGGCACCGTGCGCGCCGTGCGGCCGAGGATCGTCACCGTGGTGGAGCAGGAGGCC 1419
Rht1-B    GTCCTGGGCACCGTGCGCGCCGTGCGGCCGAGGATCGTCACCGTGGTGGAGCAGGAGGCG 1422
Rht1-D    GTCCTGGGCACCGTGCGCGCCGTGCGGCCCAGGATCGTCACCGTGGTGGAGCAGGAGGCG 1425
          ***************************** ****************************** 

Rht1-A    AACCACAACTCCGGCACATTCCTGGACCGCTTCACCGAGTCTCTGCACTACTACTCCACC 1479
Rht1-B    AACCACAACTCCGGCACATTCCTGGACCGCTTCACCGAGTCCCTGCACTACTACTCCACC 1482
Rht1-D    AATCACAACTCCGGCACATTCCTGGACCGCTTCACCGAGTCTCTGCACTACTACTCCACC 1485
          ** ************************************** ******************

Rht1-A    ATGTTCGATTCCCTCGAGGGCGGCAGCTCCGGCGGC---CCATCCGAAGTCTCATCGGGG 1536
Rht1-B    ATGTTCGATTCTCTGGAGGGCGGCAGCTCCGGCGGC---CCATCCGAAGTCTCATCTGGG 1539
Rht1-D    ATGTTCGATTCCCTCGAGGGCGGCAGCTCCGGCGGCGGCCCATCCGAAGTCTCATCGGGG 1545
          *********** ** *********************   ***************** ***

Rht1-A    GCTGCCGCTGCTCCTGCCGCCGCCGGCACGGACCAGGTCATGTCCGAGGTGTACCTCGGC 1596
Rht1-B    GCGGCTGCTGCTCCTGCCGCCGCCGGCACGGACCAGGTCATGTCCGAGGTGTACCTCGGC 1599
Rht1-D    GCTGCTGCTGCTCCTGCCGCCGCCGGCACGGACCAGGTCATGTCCGAGGTGTACCTCGGC 1605
          ** ** ******************************************************

Rht1-A    CGGCAGATCTGCAACGTGGTGGCCTGCGAGGGGGCGGAGCGCACAGAGCGCCACGAGACG 1656
Rht1-B    CGGCAGATCTGCAACGTGGTGGCCTGCGAGGGGGCGGAGCGCACAGAGCGGCACGAGACC 1659
Rht1-D    CGGCAGATCTGCAACGTGGTGGCCTGCGAGGGGGCGGAGCGCACAGAGCGCCACGAGACG 1665
          ************************************************** ******** 

Rht1-A    CTGGGGCAGTGGCGGAACCGGCTGGGCAACGCCGGGTTCGAGACCGTGCACCTGGGCTCC 1716
Rht1-B    CTGGGGCAGTGGCGGAACCGCCTCGGCAACGCCGGGTTCGAGACCGTCCACCTGGGCTCC 1719
Rht1-D    CTGGGCCAGTGGCGGAACCGGCTGGGCAACGCCGGGTTCGAGACCGTCCACCTGGGCTCC 1725
          ***** ************** ** *********************** ************

Rht1-A    AATGCCTACAAGCAGGCGAGCACGCTGCTGGCCCTATTCGCCGGCGGCGACGGGTACAAG 1776
Rht1-B    AATGCCTACAAGCAGGCGAGCACGCTGCTGGCGCTCTTCGCAGGCGGCGACGGGTACAAG 1779
Rht1-D    AATGCCTACAAGCAGGCGAGCACGCTGCTGGCGCTCTTCGCCGGCGGCGACGGCTACAAG 1785
          ******************************** ** ***** *********** ******

Rht1-A    GTGGAGGAGAAGGAGGGCTGCCTGACTCTCGGGTGGCACACGCGCCCGCTGATCGCCACC 1836
Rht1-B    GTGGAGGAGAAGGAGGGCTGCCTGACGCTGGGGTGGCACACGCGCCCGCTGATCGCCACC 1839
Rht1-D    GTGGAGGAGAAGGAAGGCTGCCTGACGCTGGGGTGGCACACGCGCCCGCTGATCGCCACC 1845
          ************** *********** ** ******************************

Rht1-A    TCGGCATGGCGCCTGGCCGCGCCGTGA 1863
Rht1-B    TCCGCATGGCGCCTGGCCGCGCCGTGA 1866
Rht1-D    TCGGCATGGCGCCTGGCCGGGCCGTGA 1872
          ** **************** *******
```

Additional Data File 3 Sequence alignment of three copies of *Rht1* gene (A, B, D copies). The regions of the gene covered by the sequenced reads from mutant and wild plants are shown by colored lines. Homoeologous SNPs are depicted in yellow whereas position of EMS induced SNPs are highlighted in cyan. Underlined SNP indicated detection both by redefined as well as first criteria whereas non-underlined SNP indicated detection with redefined criteria only.
