# Supplementary material for: Sequencing-based high throughput mutation detection in bread wheat
Source: BMC Genomics. 2015 Nov 17;16:962. doi: 10.1186/s12864-015-2112-1 (PMC4650848; doi:10.1186/s12864-015-2112-1)
Supplement: Additional file 4: — Is a figure showing sequence alignment of three copies of TaABCB1 gene. (PDF 71 kb) [file 12864_2015_2112_MOESM4_ESM.pdf]

```
TaABCB1-A   ATGTCTAGCGACCCTGAAGAGATCAAGGCTCGCGTCGTCGTCCACGGCGCCG-------- 52
TaABCB1-B   ATGTCTAGCGACCCTGAAGAGATCAAGGCTCGCGTCGTCGTCCACGGCGCCGACGCCGAC 60
TaABCB1-D   ATGTCTAGCGACCCTGAAGAGATCAAGGCTCGCGTCGTCGTCCACGGCGCCGGCGCCGAC 60
            ****************************************************

TaABCB1-A   ----ACGCCGCCGACGAGTGGGCCCGCCCGGAGCTCGAGGCCTTCCACCTCCCCTCCACC 108
TaABCB1-B   GCCGACGCCGACGCCGAGTGGGCCCGCCCGGAGCTCGAGGCCTTCCACCTCCCCTCCACC 120
TaABCB1-D   GCCAACGCCGCCGACGAGTGGGCCCGCCCGGAGCTCGAGGCCTTCCACCTCCCCTCCACC 120
                ****** ** **********************************************

TaABCB1-A   TCCCAGCCCCCACACTTGTTCCACCCACCGCACCCAGAGCCAGAAGCAGCAGAGCAATCC 168
TaABCB1-B   TCCCAGCCCCCACACTTGTTTCACCCACCGCACCCGGAGCCAGAAGCAGCAGAGCAATCC 180
TaABCB1-D   TCCCAGCCCCCGCACTTGTTCCACCCACCGCACCCAGAGCCAGAAGCAGCAGAGCAATCC 180
            *********** ******** ************** ************************

TaABCB1-A   ACGCCAGCACCGGCCGTGGCAGCAGCTACC---ACCAACAATGCGTCTGGTTCCCCCCCT 225
TaABCB1-B   ACGCCAGCACCGGCCGTCGCAGCAGCTACC---ACCAACAATGCGTCAGGTTCCCCCCCT 237
TaABCB1-D   ACGCCAGCACCGGCCGTGGCAGCAGCTACCGGCACCAACAATGCGTCTGGTTCCCCCCCT 240
            ***************** ************   ************** ************

TaABCB1-A   CCTCCCC------------CCGCGCCACTGGAGACGGAGCAGCTTCCGCCCAATGCCAAG 273
TaABCB1-B   CCTCCTCGCTCGCCGCCTCCCGCGCCACTGGAGACGGAGCAGCTTCCGCCCAATGCCAAG 297
TaABCB1-D   CCTCCTCGCTCGCCGCCTCCCGCGCCACTGGAGACGGAGCAGCTTCCGCCCAATGCCAAG 300
            ***** *            *****************************************

TaABCB1-A   CCGCCTGCCGACGAGAAGCCGCCCCCTCCGGCGCCGGCGGCCGCCCTGCGCGACTTGTTC 333
TaABCB1-B   CCGCCTGCCGACGAGAAGCCGCCCCCTCCGGCGCCGGCGGCCGCTCTGCGCGACTTGTTC 357
TaABCB1-D   CCGCCTGCCGACGAGAAGCCGCCCCCTCCGGCGCCGGCGGCCGCCCTGCGCGACTTGTTC 360
            ******************************************** ***************

TaABCB1-A   CGCTTCGCCGACGGCCTAGATCGCGTCCTCATGGCCGTGGGCACGCTCGGCGCCCTCGTC 393
TaABCB1-B   CGCTTCGCCGACGGCCTAGATCGCGTCCTCATGGCCGTGGGCACGCTCGGCGCTCTCGTC 417
TaABCB1-D   CGCTTCGCCGACGGCCTAGATCGCGTCCTCATGGCCGTGGGCACGCTCGGCGCCCTCGTC 420
            ***************************************************** ******

TaABCB1-A   CACGGCTGCTCCCTCCCCGTCTTCCTCCGCTTCTTCGCCGACCTCGTCGACTCCTTCGGC 453
TaABCB1-B   CATGGCTGCTCCCTCCCCGTCTTCCTCCGCTTCTTCGCCGACCTCGTCGACTCCTTCGGC 477
TaABCB1-D   CACGGCTGCTCCCTCCCCGTCTTCCTCCGCTTCTTCGCCGACCTCGTCGACTCCTTCGGC 480
            ** *********************************************************

TaABCB1-A   TCCCACGCCGACGACCCGGACACCATGGTCCGCCTCGTCGTGAAGTACGCCTTCTACTTC 513
TaABCB1-B   TCCCACGCCGACGACCCGGACACCATGGTCCGCCTCGTCGTCAGGTACGCCTTCTACTTC 537
TaABCB1-D   TCCCACGCCGACGACCCGGACACCATGGTCCGCCTCGTCGTCAAGTACGCCTTCTACTTC 540
            ***************************************** * ****************

TaABCB1-A   CTCGTCGTGGGCGCCGCCATCTGGGCGTCGTCCTGGGCCGAGATTTCCTGCTGGATGTGG 573
TaABCB1-B   CTCGTCGTCGGCGCCGCCATCTGGGCGTCGTCCTGGGCCGAGATTTCCTGCTGGATGTGG 597
TaABCB1-D   CTCGTCGTGGGCGCCGCCATCTGGGCGTCGTCCTGGGCCGAGATTTCCTGCTGGATGTGG 600
            ******** ***************************************************

TaABCB1-A   ACCGGCGAGCGGCAGTCCACTCGGATGCGGATCCGGTACCTCCAGGCGGCGCTCAAGCAG 633
TaABCB1-B   ACCGGCGAGCGGCAGTCCACTCGGATGCGGATCCGGTACCTCCAGGCGGCGCTCAAGCAG 657
TaABCB1-D   ACCGGCGAGCGGCAGTCCACCCGGATGCGGATCCGGTACCTCCAGGCGGCGCTCAAGCAG 660
            ******************** ***************************************

TaABCB1-A   GACGTCTCCTTCTTCGACACCGACGTGCGCACCTCCGACGTCATCTATGCCATCAACGCC 693
TaABCB1-B   GACGTCTCCTTCTTCGACACCGACGTGCGCACCTCCGACGTCATTTATGCCATCAACGCC 717
TaABCB1-D   GACGTCTCCTTCTTCGACACCGACGTGCGCACCTCCGACGTCATCTATGCCATCAACGCC 720
            ******************************************** ***************

TaABCB1-A   GACGCTGTCATGGTCCAGGACGCCATCAGCGAGAAGCTCGGCAACCTCATCCACTACATG 753
TaABCB1-B   GACGCTGTCATCGTCCAGGACGCCATCAGCGAGAAGCTCGGCAACCTCATCCACTACATG 777
TaABCB1-D   GACGCTGTCATCGTCCAGGACGCCATCAGCGAGAAGCTCGGCAACCTCATCCACTACATG 780
            *********** ************************************************

TaABCB1-A   GCCACCTTCGTCGCCGGCTTCGTCGTCGGCTTCACCGCAGCGTGGCAGCTCGCGCTCGTC 813
TaABCB1-B   GCCACCTTCGTCGCCGGCTTCGTCGTCGGCTTCACCGCAGCGTGGCAGCTCGCGCTCGTC 837
TaABCB1-D   GCCACCTTCGTGGCCGGCTTCGTCGTCGGCTTCACCGCAGCGTGGCAGCTCGCGCTCGTC 840
            *********** ************************************************

TaABCB1-A   ACGCTCGCCGTCGTGCCGCTCATCGCCGTCATCGGCGGCCTCACCGCCGCCACCATGGGC 873
TaABCB1-B   ACGCTCGCCGTCGTGCCGCTCATCGCCGTCATCGGCGGCCTCACCGCCGCCACCATGGGC 897
TaABCB1-D   ACGCTGGCCGTCGTGCCGCTCATCGCCGTCATCGGCGGCCTCACCGCCGCCACCATGGGC 900
            ***** ******************************************************

TaABCB1-A   AAGCTCTCCTCCAAGAGCCAGGACGCGCTGTCCAGCGCCAGCAACATCGCGGAGCAGGCC 933
TaABCB1-B   AAGCTCTCCTCCAAGAGCCAGGACGCGCTGTCCAGCGCCAGCAACATCGCGGAGCAGGCC 957
TaABCB1-D   AAGCTCTCCTCCAAGAGCCAGGACGCGCTGTCCAGCGCCAGCAACATCGCGGAGCAGGCC 960
            ************************************************************

TaABCB1-A   CTGTCGCAGATACGAGTCGTGCAGTCGTTCGTGGGTGAGGAGCGGGTGGCGCAGGCCTAC 993
TaABCB1-B   CTGTCGCAGATACGGATCGTGCAGTCATTCGTGGGTGAGCAGCGGGTGGCGCAGGCCTAC 1017
TaABCB1-D   CTGTCGCAGATACGGATCGTGCAGTCGTTCGTGGGTGAGCAGCGGGTGGCGCAGGCCTAC 1020
            **************  ********** ************ ********************
```

```
TaABCB1-A   TCGGCGGCGCTAGCCGTGGCGCAGAGCATCGGCTACCGGAACGGCTTTGCCAAGGGCCTC 1053
TaABCB1-B   TCGGCGGCGCTAGCCGTGGCGCAGAGCATCGGCTACCGGAACGGCTTTGCCAAGGGCCTC 1077
TaABCB1-D   TCGGAGGCGCTAGCCGTGGCGCAGAGCATCGGCTACCGGAACGGCTTTGCCAAGGGCCTC 1080
            **** *******************************************************

TaABCB1-A   GGGCTGGGCGGCACCTACTTCACCGTCTTCTGCTGCTACGCTCTGCTCCTCTGGTACGGC 1113
TaABCB1-B   GGGCTGGGCGGCACCTACTTCACCGTCTTCTGCTGCTACGCTCTGCTCCTCTGGTACGGC 1137
TaABCB1-D   GGGCTGGGCGGCACCTACTTCACCGTCTTCTGCTGCTACGCTCTGCTCCTCTGGTACGGC 1140
            ************************************************************

TaABCB1-A   GGGCACCTCGTTCGCGGCCACCACACCAACGGCGGGCTGGCCATCGCCACCATGTTCTCC 1173
TaABCB1-B   GGGCACCTCGTTCGCGGCCACCACACCAACGGCGGGCTGGCCATCGCCACCATGTTTTCC 1197
TaABCB1-D   GGGCACCTCGTTCGCGGCCACCACACCAACGGCGGGCTGGCCATCGCCACCATGTTCTCC 1200
            ******************************************************** ***

TaABCB1-A   GTCATGATCGGCGGACTCGGTCTTGGGCAGTCGGCGCCGAGCATGGCGGCGTTTGCCAAG 1233
TaABCB1-B   GTCATGATCGGCGGACTCGCTCTTGGGCAGTCGGCGCCGAGCATGGCGGCGTTTGCCAAG 1257
TaABCB1-D   GTCATGATCGGCGGACTCGGTCTTGGGCAGTCGGCGCCGAGCATGGCGGCGTTTGCCAAG 1260
            ******************* ****************************************

TaABCB1-A   GCGAGGGTTGCGGCCGCGAAGATCTTCCGTATCATCGACCACACGCCAGGCATCACCAAA 1293
TaABCB1-B   GCGAGGGTTGCGGCCGCGAAGATCTTCCGTATCATCGACCACACGCCAGGCGTCACCAAG 1317
TaABCB1-D   GCGAGGGTTGCGGCCGCGAAGATCTTCCGTATCATCGACCACACGCCAGGCATCACCAAG 1320
            *************************************************** ******* 

TaABCB1-A   GAAGGCGTAGAGCTGGAGTCGGTGACCGGGCGTCTGGAGCTGAGGAACGTGGAGTTCGCG 1353
TaABCB1-B   GAAGGCGTGGAGCTGGAGTCGGTGACCGGGCGGCTGGAGCTGAGGAACGTGGAGTTCGCG 1377
TaABCB1-D   GAAGGCGTGGAGCTGGAGTCGGTGACGGGGCGGCTGGAGCTGAGGAACGTGGAGTTCGCG 1380
            ******** ***************** ***** ***************************

TaABCB1-A   TACCCGTCCCGGCCGGACACGCCGATTCTGCGCCGCTTCTCTCTAAGCGTACCGGCCGGG 1413
TaABCB1-B   TACCCGTCCCGGCCGGACACGCCGATTCTGCGCCGCTTCTCTCTAAGCGTACCGGCAGGG 1437
TaABCB1-D   TACCCGTCCCGGCCGGACACGCCGATTCTGCGCCGCTTCTCTCTAAGCGTACCGGCCGGG 1440
            ******************************************************** ***

TaABCB1-A   AAGACGATCGCTCTGGTTGGCAGCTCTGGCTCCGGGAAGAGTACGGTGGTGTCCCTGATC 1473
TaABCB1-B   AAGACGATCGCTCTGGTTGGCAGCTCTGGCTCCGGGAAGAGTACGGTGGTGTCCCTGATC 1497
TaABCB1-D   AAGACGATCGCTCTGGTTGGCAGCTCTGGCTCCGGGAAGAGTACGGTGGTGTCGCTGATC 1500
            ***************************************************** ******

TaABCB1-A   GAGAGGTTCTACGACCCGAGTTCAGGGCAAATCATGCTTGACGGTGTCGAGCTCAAGGAT 1533
TaABCB1-B   GAGAGGTTCTACGACCCGAGTTCAGGGCAAATCATGCTTGACGGTGTTGAGCTCAAGGAT 1557
TaABCB1-D   GAGAGGTTCTACGACCCGAGTTCAGGGCAAATCATGCTTGACGGTGTTGAGCTCAAGGAT 1560
            *********************************************** ************

TaABCB1-A   CTGAAGCTGCGGTGGCTGCGGTCGCAGATCGGCCTGGTGAGCCAGGAGCCGGCGCTGTTC 1593
TaABCB1-B   CTGAAGCTGCGGTGGCTGCGGTCGCAGATCGGCCTGGTGAGCCAGGAGCCGGCGCTGTTC 1617
TaABCB1-D   CTGAAGCTGCGGTGGCTGCGGTCGCAGATCGGCCTGGTGAGCCAGGAGCCGGCGCTGTTC 1620
            ************************************************************

TaABCB1-A   GCGACGAGCATCAGGGAGAACCTGCTGCTTGGGAGGGAGGAGGCGAGCCAGGTGGAGATG 1653
TaABCB1-B   GCGACGAGCATCAGGGAGAACCTGCTGCTTGGGAGGGAGGAGGCGAGCCAGGTGGAGATG 1677
TaABCB1-D   GCGACGAGCATCAGGGAGAACCTGCTGCTTGGGAGGGAGGAGGCGAGCCAGGTGGAGATG 1680
            ************************************************************

TaABCB1-A   GAGGAGGCCGCCAGGGTCGCCAACGCCCACTCCTTCATCATCAAGCTCCCCGACGGCTAC 1713
TaABCB1-B   GAGGAGGCCGCCAGGGTCGCCAACGCCCACTCCTTCATCATCAAGCTCCCCGACGGCTAC 1737
TaABCB1-D   GAGGAGGCCGCCAGGGTCGCCAACGCCCACTCCTTCATCATCAAGCTCCCCGACGGCTAC 1740
            ************************************************************

TaABCB1-A   GACACGCAGGGTGGGGGAGCGCGGCCTGCAGCTCTCCGGCGGCCAGAAGCAGCGGATCGC 1773
TaABCB1-B   GACACGCAGG-TGGGGGAGCGCGGCCTGCAGCTCTCCGGCGGCCAGAGGCAGCGGATCGC 1796
TaABCB1-D   GACACGCAGGGTGGGGGAGCGCGGCCTGCAGCTCTCCGGCGGCCAGAAGCAGCGGATCGC 1800
            ********** ************************************ ************

TaABCB1-A   CATCGCCCGTGCGATGCTCAAGAACCCGGCCATCCTCCTCCTGGACGAGGCCACCAGCGC 1833
TaABCB1-B   CATTGCCCGTGCGATGCTCAAGAACCCGGCCATCCTCCTCCTGGACGAGGCCACCAGCGC 1856
TaABCB1-D   CATTGCCCGTGCGATGCTCAAGAACCCGGCCATCCTCCTCCTGGACGAGGCCACCAGCGC 1860
            *** ********************************************************

TaABCB1-A   GCTGGACTCCGAGTCCGAGAAGCTCGTGCAGGAGGCGCTGGACCGCTTCATGATCGGCCG 1893
TaABCB1-B   GCTGGACTCCGAGTCGGAGAAGCTCGTGCAGGAGGCGCTGGACCGCTTCATGATCGGCCG 1916
TaABCB1-D   GCTGGACTCCGAGTCGGAGAAGCTCGTGCAGGAGGCGCTGGACCGCTTCATGATCGGCCG 1920
            *************** ********************************************

TaABCB1-A   CACCACCCTTGTCATCGCGCACAGGCTCTCAACCATCCGCAAGGCCGACCTCGTTGCCGT 1953
TaABCB1-B   CACCACCCTCGTCATCGCGCACAGGCTCTCCACCATCCGCAAGGCCGACCTCGTCGCCGT 1976
TaABCB1-D   CACCACCCTGGTCATCGCGCACAGGCTCTCCACCATCCGCAAGGCCGACCTCGTCGCCGT 1980
            ********* ******************** *********************** *****

TaABCB1-A   CCTGCAGGCTGGCGCCGTGTCCGAGATGGGCGCGCACGACGACCTCATGGCCAGAGGGGA 2013
TaABCB1-B   CCTGCAGGCTGGCGCCGTCTCCGAGATGGGCGCGCACGACGACCTCATGGCCAGAGGGGA 2036
TaABCB1-D   CCTGCAGGCTGGCGCCGTGTCCGAGATGGGCGCGCACGACGATCTCATGGCCAGAGGGGA 2040
            ****************** *********************** *****************

TaABCB1-A   CAGCGGCGCGTACGCCAAGCTCATCCGCATGCAGGAGCAGGCGCACGAGGCGGCCATCGT 2073
TaABCB1-B   CAGCGGCGCGTACGCCAAGCTCATCCGCATGCAGGAGCAGGCGCACGAGGCGGCCCTCGT 2096
```

```
TaABCB1-D   CAGCGGCGCGTACGCCAAGCTCATCCGCATGCAGGAGCAGGCGCACGAGGCGGCCCTCGT 2100
            ******************************************************* ****

TaABCB1-A   CAGCGCCAGGAGGAGCAGCGCAAGGCCCTCCAGCGCCCGCAACTCCGTCAGCTCACCCAT 2133
TaABCB1-B   CAGCGCCAGGAGGAGCAGCGCAAGGCCCTCCAGCGCCCGCAACTCCGTCAGCTCACCCAT 2156
TaABCB1-D   CAGCGCTAGGAGGAGCAGCGCAAGGCCCTCCAGCGCCCGCAACTCCGTCAGCTCACCCAT 2160
            ****** *****************************************************

TaABCB1-A   CATGATGCGCAACTCCTCCTACGGCCGCTCGCCCTACTCCCGCCGCCTCTCCGACTTCTC 2193
TaABCB1-B   CATGATGCGCAACTCCTCCTACGGCCGCTCGCTCTACTCCCGCCGCCTCTCCGACTTCTC 2216
TaABCB1-D   CATGATGCGCAACTCCTCCTACGGCCGCTCGCCCTACTCCCGCCGCCTCTCCGACTTCTC 2220
            ******************************** ***************************

TaABCB1-A   CACCGCCGACTTCAGCCTGTCCGTCATACATGACCCGGCCGCCCACCGGATGGGCATGGG 2253
TaABCB1-B   CACCGCCGACTTCAGCCTGTCCGTCATACATGACCCGGCCGCCCACCGGATGGGCAT--- 2273
TaABCB1-D   CACCGCCGACTTCAGCCTGTCCGTCATACATGACCCAGCCGCCCACCGGATGGGCATGGG 2280
            ************************************ ********************   

TaABCB1-A   CATGGGAATGG------AGAAGCTCGCGTTCCGTGCGCAGGCCAGCTCCTTCTGGCGGCT 2307
TaABCB1-B   ---GGGAATGG------AGAAGCTCGCGTTCCGTGCGCAGGCCAGCTCCTTCTGGCGGCT 2324
TaABCB1-D   CATGGGAATGGGAATGGAGAAGCTGGCGTTCCGTGCGCAGGCCAGCTCCTTCTGGCGGCT 2340
               ********      ******* ***********************************

TaABCB1-A   GGCCAAGATGAACTCGCCCGAGTGGGGCTACGCGCTCGCCGGCTCCGTGGGGTCCATGGT 2367
TaABCB1-B   GGCCAAGATGAACTCGCCGGAGTGGGGCTACGCGCTCGCCGGCTCCGTGGGGTCCATGGT 2384
TaABCB1-D   GGCCAAGATGAACTCGCCCGAGTGGGGCTACGCGCTCGCCGGCTCCGTGGGGTCCATGGT 2400
            ****************** *****************************************

TaABCB1-A   GTGCGGCTCCTTCAGCGCTATCTTCGCTTACATCCTCAGCGCGGTGCTCAGCATCTACTA 2427
TaABCB1-B   GTGCGGCTCCTTCAGCGCCATCTTCGCCTACATCCTCAGCGCGGTGCTCAGCATCTACTA 2444
TaABCB1-D   ATGCGGCTCCTTCAGCGCCATCTTCGCCTACATCCTCAGCGCGGTGCTCAGCATCTACTA 2460
             ***************** ******** ********************************

TaABCB1-A   CACGCCGGACCCGAGGCACATGGACCGGGAGATCGCCAAGTACTGCTACCTCCTCATCGG 2487
TaABCB1-B   CACGCCGGACCCGAGGCACATGGACCGGGAGATCACCAAGTACTGCTACCTCCTCATCGG 2504
TaABCB1-D   CACGCCGGACCCGAGGCACATGGACCGGGAGATCGCCAAGTACTGCTACCTCCTCATCGG 2520
            ********************************** *************************

TaABCB1-A   CATGTCCTCTGCCGCGCTGCTCTTCAACACCGTGCAGCACCTCTTCTGGGACACGGTGGG 2547
TaABCB1-B   CATGTCCTCCGCCGCGCTGCTCTTCAACACCGTGCAGCACCTGTTCTGGGACACGGTGGG 2564
TaABCB1-D   CATGTCCTCCGCCGCGCTGCTCTTCAACACCGTGCAGCACCTCTTCTGGGACACGGTGGG 2580
            ********* ******************************** *****************

TaABCB1-A   CGAGAACCTCACCAAGCGCGTGCGCGAGAAGATGCTCACGGCGGTGCTCCGCAACGAGAT 2607
TaABCB1-B   CGAGAACCTCACCAAGCGCGTGCGCGAGAAGATGCTCACGGCGGTGCTCCGCAACGAGAT 2624
TaABCB1-D   CGAGAACCTCACCAAGCGCGTGCGCGAGAAGATGCTCACGGCGGTGCTCCGCAACGAGAT 2640
            ************************************************************

TaABCB1-A   GGCCTGGTTCGACATGGAGGCCAATGCCAGCGCGCACATCGCTGCCAGGCTGGCGCTGGA 2667
TaABCB1-B   GGCCTGGTTCGACATGGAGGCCAATGCGAGTGCGCACATCGCTGCCAGGCTCGCGCTGGA 2684
TaABCB1-D   GGCCTGGTTCGACATGGAGGCCAATGCCAGCGCGCACATCGCTGCCAGGCTCGCGCTGGA 2700
            *************************** ** ********************* *******

TaABCB1-A   CGCCCAGAACGTGCGCTCCGCCATCGGGGACCGCATCTCCATCATCGTGCAGAACTCGGC 2727
TaABCB1-B   CGCCCAGAACGTGCGCTCCGCCATCGGGGACCGCATCTCCATCATCGTGCAGAACTCGGC 2744
TaABCB1-D   CGCCCAGAACGTGCGCTCCGCCATCGGGGACCGCATCTCCATCATCGTGCAGAACTCGGC 2760
            ************************************************************

TaABCB1-A   GCTTATGCTCGTCGCATGCACGGCCGGGTTCGTCCTGCAGTGGCGCCTCGCGCTCGTGCT 2787
TaABCB1-B   GCTTATGCTCGTCGCATGCACGGCCGGGTTCGTCCTGCAGTGGCGCCTCGCGCTCGTGCT 2804
TaABCB1-D   GCTTATGCTCGTCGCCTGCACGGCCGGGTTCGTCCTGCAGTGGCGCCTCGCGCTCGTGCT 2820
            *************** ********************************************

TaABCB1-A   CCTCGCCGTCTTCCCACTCGTCGTCGGCGCCACCGTCCTGCAGAAGATGTTCATGAAGGG 2847
TaABCB1-B   CCTCGCCGTCTTCCCACTCGTCGTCGGCGCCACCGTCCTGCAGAAGATGTTCATGAAGGG 2864
TaABCB1-D   CCTCGCCGTCTTCCCACTCGTCGTCGGCGCCACCGTCCTGCAGAAGATGTTCATGAAGGG 2880
            ************************************************************

TaABCB1-A   TTTCTCGGGTGACCTGGAAGGCGCGCACGCCAAGGCGACGCAGATTGCGGGGGAGGCGGT 2907
TaABCB1-B   TTTCTCGGGTGACCTGGAAGGCGCGCACGCCAAGGCGACGCAGATTGCGGGGGAGGCTGT 2924
TaABCB1-D   TTTCTCGGGTGACCTGGAAGCCGCGCACGCCAAGGCGACGCAAATTGCGGGGGAGGCGGT 2940
            ******************** ********************* ************** **

TaABCB1-A   GGCCAACGTGCGCACCGTGGCGGCGTTCAACTCGGAGGACAAGATCACGAGGCTCTTCGA 2967
TaABCB1-B   GGCCAACGTGCGCACCGTGGCGGCGTTCAACTCGGAGGACAAGATCACGAGGCTCTTCGA 2984
TaABCB1-D   GGCCAACGTGCGCACCGTGGCGGCGTTCAACTCGGAGGACAAGATCACGAGGCTCTTCGA 3000
            ************************************************************

TaABCB1-A   GGCCAACCTGCAGAGGCCGCTCCGTCGCTGCTTCTGGAAGGGCCAGATCGCGGGCATCGG 3027
TaABCB1-B   GGCCAACCTGCAGAGGCCGCTCCGGCGCTGCTTCTGGAAGGGCCAGATCGCCGGCATCGG 3044
TaABCB1-D   GGCCAACCTGCAGAGGCCGCTCCGGCGCTGCTTCTGGAAGGGCCAGATCGCCGGCATCGG 3060
            ************************ ************************** ********

TaABCB1-A   CTACGGTGTGGCGCAGTTCCTGCTGTACGCGTCCTACGCGCTGGGCCTGTGGTACGCTGC 3087
TaABCB1-B   CTACGGTGTGGCGCAGTTCCTGCTGTACGCGTCCTACGCGCTGGGCCTGTGGTACGCTGC 3104
TaABCB1-D   CTACGGTGTGGCGCAGTTCCTGCTGTACGCGTCCTACGCGCTGGGCCTGTGGTACGCTGC 3120
            ************************************************************
```

```
TaABCB1-A   GTGGCTGGTGAAGCACGGCATCTCCGACTTCTCCAAGACCATCCGCGTCTTCATGGTGCT 3147
TaABCB1-B   GTGGCTGGTGAAGCACGGCATCTCCGACTTCTCCAAGACCATCCGCGTCTTCATGGTGCT 3164
TaABCB1-D   GTGGCTGGTGAAGCACGGCGTCTCCGACTTCTCCAAGACCATCCGCGTCTTCATGGTGCT 3180
            ******************* ****************************************

TaABCB1-A   CATGGTCTCCGCCAACGGTGCCGCCGAGACGCTGACGCTGGCGCCGGACTTCATCAAGGG 3207
TaABCB1-B   CATGGTCTCCGCCAACGGTGCCGCTGAGACGCTGACGCTGGCGCCGGACTTCATCAAGGG 3224
TaABCB1-D   CATGGTCTCCGCCAACGGTGCCGCCGAGACGCTGACGCTGGCGCCGGACTTCATCAAGGG 3240
            ************************ ***********************************

TaABCB1-A   CGGGCGGGCCATGCAGTCGGTGTTCGAGACCATCGACCGCAAGACGGAGATCGAGCCCGA 3267
TaABCB1-B   CGGGCGGGCCATGCAGTCGGTGTTCGAGACCATCGACCGCAAGACGGAGATCGAGCCCGA 3284
TaABCB1-D   CGGGCGGGCCATGCAGTCGGTGTTCGAGACCATCGACCGCAAGACGGAGATCGAGCCCGA 3300
            ************************************************************

TaABCB1-A   CGACGTGGACGCCGCAGCAGTCCCCGAGCGTCCCAGGGGCGACGTGGAGTTGAAGCACGT 3327
TaABCB1-B   CGACGTGGACGCCGCAGCCGTCCCCGAGCGGCCCAGGGGCGACGTGGAGTTGAAGCACGT 3344
TaABCB1-D   CGACGTGGACGCCGCAGCCGTCCCCGAGCGGCCCAGGGGCGACGTGGAGTTGAAGCACGT 3360
            ****************** *********** *****************************

TaABCB1-A   CGACTTCTCGTACCCGTCGCGGCCGGACGTTCAGGTGTTCCGGGACCTGAGCCTCCGCGC 3387
TaABCB1-B   CGACTTCTCGTACCCGTCGCGGCCGGACATTCAGGTGTTCCGGGACCTGAGCCTCCGCGC 3404
TaABCB1-D   CGACTTCTCGTACCCGTCGCGGCCGGACGTTCAGGTGTTCCGGGACCTGAGCCTCCGCGC 3420
            **************************** *******************************

TaABCB1-A   CCGCGCAGGCCGGACGCTGGCGCTGGTGGGGCCCAGCGGGTGCGGCAAGAGCTCCGTGCT 3447
TaABCB1-B   GCGCGCAGGCCGGACGCTGGCGCTGGTGGGGCCCAGCGGGTGCGGCAAGAGCTCCGTGCT 3464
TaABCB1-D   CCGCGCAGGCCGGACGCTGGCGCTGGTGGGGCCCAGCGGGTGCGGCAAGAGCTCCGTGCT 3480
             ***********************************************************

TaABCB1-A   GGCGCTCATCCAGCGCTTCTACGAGGCCAGCTCCGGGCGCGTGCTCCTGGACGGCAAGGA 3507
TaABCB1-B   GGCGCTCATCCAGCGCTTCTACGAGCCCAGCTCCGGGCGCGTGCTCCTGGACGGCAAGGA 3524
TaABCB1-D   GGCGCTCATCCAGCGCTTCTACGAGCCCAGCTCCGGGCGCGTGCTCCTGGACGGCAAGGA 3540
            ************************* **********************************

TaABCB1-A   CATCCGCAAGTACAACCTCAAGGCGCTGCGGCGAGTGGTGGCCATGGTGCCGCAGGAGCC 3567
TaABCB1-B   CATCCGCAAGTACAACCTCAAGGCGCTGCGGCGAGTGGTGGCCATGGTGCCGCAGGAGCC 3584
TaABCB1-D   CATCCGCAAGTACAACCTCAAGGCGCTGCGGCGAGTGGTGGCCATGGTGCCGCAGGAGCC 3600
            ************************************************************

TaABCB1-A   GTTTCTTTTCGCCGGCACCATCCACGACAACATCGCCTACGGGCGCGAGGGCGCGACCGA 3627
TaABCB1-B   GTTTCTTTTCGCCGGCACCATCCACGACAACATCGCCTACGGGCGCGAGGGCGCGACCGA 3644
TaABCB1-D   GTTTCTTTTCGCCGGCACCATCCACGACAACATCGCCTACGGCCGCGAGGGCGCTACCGA 3660
            ****************************************** *********** *****

TaABCB1-A   GGCGGAGGTGGTGGAGGCGGCAACGCAGGCCAACGCGCACAAGTTCGTGTCGGCGCTGCC 3687
TaABCB1-B   GGCGGAGGTGGTGGAGGCGGCAACGCAGGCCAACGCGCACAAGTTCGTGTCGGCGCTGCC 3704
TaABCB1-D   GGCGGAGGTGGTGGAGGCGGCAACGCAGGCCAACGCGCACAAGTTCGTGTCGGCGCTGCC 3720
            ************************************************************

TaABCB1-A   GGAAGGGTACAAGACGTGCGTCGGGGAGCGCGGGGTGCAGCTGTCGGGAGGGCAGCGCCA 3747
TaABCB1-B   GGAAGGGTACAAGACGTGCGTCGGGGAGCGCGGGGTGCAGCTGTCGGGAGGGCAGCGGCA 3764
TaABCB1-D   GGAAGGGTACAAGACGTGCGTCGGGGAGCGCGGGGTGCAGCTGTCGGGAGGGCAGCGCCA 3780
            ********************************************************* **

TaABCB1-A   GCGGATCGCCATCGCGCGAGCGCTGGTGAAGCAGGCGGCCATCATGCTGCTGGACGAGGC 3807
TaABCB1-B   GCGGATCGCCATCGCGCGAGCGCTGGTGAAGCAGGCGGCCATCATGCTGCTGGACGAGGC 3824
TaABCB1-D   GCGGATCGCCATCGCGCGAGCGCTGGTGAAGCAGGCGGCCATCATGCTGCTGGACGAGGC 3840
            ************************************************************

TaABCB1-A   GACGAGTGCGCTGGACGCCGAGTCTGAGCGGTGCGTGCAGGAGGCGCTGGACCGGGCCGG 3867
TaABCB1-B   GACGAGTGCCCTGGACGCCGAGTCGGAGCGGTGCGTGCAGGAGGCGCTGGACCGGGCCGG 3884
TaABCB1-D   GACGAGTGCGCTGGACGCCGAGTCGGAGCGGTGCGTGCAGGAGGCGCTGGACCGGGCCGG 3900
            ********* ************** ***********************************

TaABCB1-A   GCCAGGGTCAGGGCGAACGACCATCGTGGTGGCGCACCGCCTGGCCACGGTGCGGAACGC 3927
TaABCB1-B   GTCAGGGTCAGGGCGAACGACCATCGTGGTGGCGCACCGGCTGGCCACGGTGCGGAACGC 3944
TaABCB1-D   GTCAGGGTCAGGGCGAACGACCATCGTGGTGGCGCACCGCCTGGCCACGGTGCGGAACGC 3960
            * ************************************* ********************

TaABCB1-A   GCACACCATCGCGGTCATCGACGACGGCAAGGTGGTGGAGCAAGGGTCGCACTCGCACCT 3987
TaABCB1-B   GCACACCATCGCGGTGATCGACGACGGCAAGGTGGTGGAGCAAGGGTCGCACTCGCATCT 4004
TaABCB1-D   GCACACCATCGCGGTCATCGACGACGGCAAGGTGGTGGAGCAAGGGTCGCACTCGCACCT 4020
            *************** ***************************************** **

TaABCB1-A   GCTCAACCATCACCCCGACGGATGCTACGCGCGGATGCTGCAGCTGCAGCGCCTCACGCC 4047
TaABCB1-B   GCTCAACCATCACCCCGACGGATGCTACGCGCGGATGCTGCAGTTCCAGCGCCTCACGCC 4064
TaABCB1-D   GCTCAACCATCACCCCGACGGATGCTACGCGCGGATGCTGCAGCTGCAGCGCCTCACGCC 4080
            ******************************************* * **************

TaABCB1-A   CCACACCCTTGCCGTGCCCGGACCCGGACCCGGATCCTCTGCATCCAACGTT 4099
TaABCB1-B   CCACACCCTTGCCGTGCCCGGAC------------CCTCTGCATCCAACGTT 4104
TaABCB1-D   CCACACCCTTGCCGTGCCCGGACCCGGAC------CCTCTGCATCCAACGTT 4126
            ***********************            ****************
```

**Additional Data File 4** Sequence alignment of three copies of *TaABCB1* gene (A, B, D copies). The regions of the gene covered by the sequenced reads from mutant and wild plants are shown by colored lines. Homoeologous SNPs are depicted in yellow whereas position of EMS induced SNPs are highlighted in cyan. Underlined SNP indicated detection both by redefined as well as first criteria whereas non-underlined SNP indicated detection with redefined criteria only.
