# Supplementary material for: Sequencing-based high throughput mutation detection in bread wheat
Source: BMC Genomics. 2015 Nov 17;16:962. doi: 10.1186/s12864-015-2112-1 (PMC4650848; doi:10.1186/s12864-015-2112-1)
Supplement: Additional file 5: — Is a figure showing sequence alignment of three copies of C-Ph1 gene. (PDF 45 kb) [file 12864_2015_2112_MOESM5_ESM.pdf]

```
C-Ph1-A    ATGGCGCGCCTCCTCGTTCTCGCCGTCACGGCCACGGTTCTCATGGGCTCGGTCCGGGCA 60
C-Ph1-B    ATGGCGCGCCTCCTCGTTCTCGCCGTCACGGCCACGGTTCTCATGG-TGCGAAAGAGCTA 59
C-Ph1-D    ATGGCGCGCCTCCTCGTTCTCGCCGTCACGGCCACGGTTCTCATGG-CTCAATCCGGGCA 59
           **********************************************   *      *  *

C-Ph1-A    GCCGGC---GTCCGC--TGCGTGG------------------ACG--------------- 82
C-Ph1-B    GCTAGTAGAGCCGGCCATGCATGGCACAGATACCTATCATACACGACTGAATTTTGTCTT 119
C-Ph1-D    GCCGGC---GTCTGC--TGCGCGG------------------ACG--------------- 81
           **  *    * * **  ***  **                  ***

C-Ph1-A    ----------TTGCCGGCGG-----------------AAGCCT----------------- 98
C-Ph1-B    GTCAAACATTTCATCGGTGCGTTTTTTTTGTCTCTCCAGGCTCAATCCGGGCAGCCGGCG 179
C-Ph1-D    ----------TCGCCGGCGG-----------------AGGCCT----------------- 97
                     *   *** *                  * **

C-Ph1-A    TCTG--GCGCGCCACCCTGCCCGACGCCCCCATGCCAGACGCCATCCTCGAGCTCCAGCC 156
C-Ph1-B    TCTGCTGCGCGCCGCCCTGCCCGACGCCCCCATGCCGGACGCCATCCTCGAGCTCCTGCC 239
C-Ph1-D    TCTG--GCGCGCCGCCCTGCCCGACGCCACCATGCCGGACGCCATCCTCGAGCTCCTGCC 155
           ****  ******* ************** ******* ******************* ***

C-Ph1-A    CCAGTTTGATCACCACACATCAACGGAACAGGG--------------------------- 189
C-Ph1-B    CCANTTTGATCACCACGCATCAACGGAACAGGGTACTGATTTTGTGGCCATCTTCCGATG 299
C-Ph1-D    CCAGGGTGATCACCACGCATCAACGGAACAGGG--------------------------- 188
           ***   ********** ****************

C-Ph1-A    -------------------------CGCATAC--------ATGAATGCAGAGACTGGCAC 216
C-Ph1-B    GAAGGCCGTCTCTCTCTAGCTCACCCACGTGCCTTGCTGCATGAATGCAGAGAAAGACAC 359
C-Ph1-D    -------------------------CGCGTAC--------ATGAATGCAGAGACCGACAC 215
                                    * * * *        *************  * ***

C-Ph1-A    TCCGGAAGGCGCAGTCGCCGAGGACAGCGTGGAGGACAACGACCCGCCGCGGCCCATGAA 276
C-Ph1-B    TCCGGAAGGCGCGGTCG---AGGAC---GTGGAGGACAAGGACCCGCCGCCGCCCATGAA 413
C-Ph1-D    ACCGGAAGGCGCGGTCG---AGGCC---GTGGAGGACAAGGACCCGCCGCCGCCCATGAA 269
            *********** ****   *** *   *********** ********** *********

C-Ph1-A    CTTCAACTACGGCTACAACAACGCCTTGCCCCGGAGCGAAGCCACCAGCGCCCCCTCCCC 336
C-Ph1-B    CTTCAACTACGACTACGATGACGCCTTGCCCCGGAGCGAAACCACCAGCGCCCCCTCCCC 473
C-Ph1-D    CTTCAACTACGACTACGACGACGCCTTGCCCCGGAGCGAAGCCACCAGCGCCCCCTCCCC 329
           *********** **** *  ******************** *******************

C-Ph1-A    CAACGTCCTACTGAACCGCGCCGCCGTCGTCCGCAACGTCGCCACGCCGTCGTCGGCGGT 396
C-Ph1-B    CGACGTCCTACTGAACCGCGCCGCCGTCGTCCGCAACGTCGCCACGCCGTCGTCGGCGGT 533
C-Ph1-D    CGACGTCCTACTAAACCGCGCTGCCGTCGTC------------ACGCCGTCGTCGACGGT 377
           * ********** ******** *********            ************ ****

C-Ph1-A    GTTCTTCCTCGAGGACGCG----------------------------------------- 415
C-Ph1-B    GTTCTTCCTCGAGGACGCGGTGCGCGTCCGGGAGAGCCTGCCCTTCCACAGGATCCATCG 593
C-Ph1-D    GTTCTTCCTCGAGGACGCGGTGCGCGTCGGGGAGAGCCTGCCCTTCCACAGGATCCATCG 437
           *******************

C-Ph1-A    ------------------------------------------------------------
C-Ph1-B    GGCCACCGGCGCTGCCGAGGCGTCGGCAGAACAGCCGCTGGAGCTGTACACTGTGCACTC 653
C-Ph1-D    GGCCACCGCCGCCGCCGAGGCGTCGGCAGAGCAGCCGCTGGAGCTGTACACCGTCCGCTC 497

C-Ph1-A    ------------------------------------------------------------
C-Ph1-B    CGTGAGGGCGGTCGAGGGGTCCAATTTCATCCTGTGCCGGGGTGAAGCCGGCGAAGGGGC 713
C-Ph1-D    CGTGAGGGCGGTCGAGGGGTCCAGTTTCGTCCTGTGCCGGGGAGAAGCCGGCGAAGGGGC 557

C-Ph1-A    ------------------------------------------------------------
C-Ph1-B    CGTGTACGGGTGCCGCGCAACCGGCCCGGCGAGGGCCTACGTCCTGGCCCTGGCCGGCGA 773
C-Ph1-D    CGTGTACGGGTGCCGCGCCACCGGCCCGGCGAGGGCCTACGTCCTGGCCCTGGCCGGCGA 617

C-Ph1-A    ------------------------------------------------------------
C-Ph1-B    GCGCGGGGACGTGACGATGACCGCGGTTGCCGTGTGCCGCACCGACGCATCCCGATGGGA 833
C-Ph1-D    GCGCGGGGACGCGGCGATGACCGCGGTTGCCGTGT----------------CCCGGTGGGA 662

C-Ph1-A    ------------------------------------------------------------
C-Ph1-B    CCCGGAGCACGCCGCCTTCCGGCTCCTGGGCGTGAAGCCCGGCGGCGCGGCGGTCTGCCA 893
C-Ph1-D    CCCGGAGCACGCCGCCTTCCGGCTCCTCGGCGTGAAGCCCGGCGGCGCGGCGGTCTGCCA 722

C-Ph1-A    --------------------------------------------------------AGTA
C-Ph1-B    CGCGGTGCGGGACGCGCAGCTCCTGCCGGCCATGAACGGGAAGAGCCCCGTCGCCAACTA 953
C-Ph1-D    CGCCGTGCCGGACGCGCAGGTCCTGCCGGCCATGAACGGGAAGAGTCCCGCCGCCAACTA 782

C-Ph1-A    A 420
C-Ph1-B    A 954
C-Ph1-D    A 783
```

Additional data file 5 Sequence alignment of three copies of *C-Ph1* gene (A, B, D copies). The regions of the gene covered by the sequenced reads from mutant and wild plants are shown by colored lines. Homoeologous SNPs are depicted in yellow whereas position of EMS induced SNPs are highlighted in cyan. Underlined SNP indicated detection both by redefined as well as first criteria whereas non-underlined SNP indicated detection with redefined criteria only.
