# Supplementary material for: Sequencing-based high throughput mutation detection in bread wheat
Source: BMC Genomics. 2015 Nov 17;16:962. doi: 10.1186/s12864-015-2112-1 (PMC4650848; doi:10.1186/s12864-015-2112-1)
Supplement: Additional file 6: — Is a table listing rice coding sequences (including and excluding TE) and cDNA with wheat unigenes at different e-values. (PDF 35 kb) [file 12864_2015_2112_MOESM6_ESM.pdf]

Additional data file 6 Comparison of rice coding sequences (including and excluding TE) and cDNA with wheat unigenes at different e-values

E-value (1e-)	CDS + TE		CDS - TE		cDNA	
	Rice	Wheat	Rice	Wheat	Rice	Wheat
80	20,129	28,581	18,693	28,033	21,798	28,216
70	21,249	31,096	19,558	30,475	22,961	30,703
60	22,446	34,052	20,494	33,340	24,180	33,630
50	23,876	37,636	21,554	36,795	25,526	37,172
40	25,554	41,811	22,728	40,787	26,952	41,308
30	27,704	47,050	24,113	45,754	28,520	46,429
20	30,720	53,806	25,752	52,027	30,223	53,160
10	34,532	62,450	27,670	61,070	31,980	62,951
