# Supplementary material for: Sequencing-based high throughput mutation detection in bread wheat
Source: BMC Genomics. 2015 Nov 17;16:962. doi: 10.1186/s12864-015-2112-1 (PMC4650848; doi:10.1186/s12864-015-2112-1)
Supplement: Additional file 7: — Is a table showing mutation rate for different mutant plants. (PDF 33 kb) [file 12864_2015_2112_MOESM7_ESM.pdf]

Additional data file 9 Mutation rate (Kb/mutation) for the mutant plants.

Mutant	Unigenes coverage (bp)	EMS SNPs	Kb/mutation
13	1510282	290	5.2
14	2093074	351	6.0
16	4314695	838	5.1
17	4530881	776	5.8
18	2987628	528	5.7
19	5110010	970	5.3
20	3156173	594	5.3
21	3264895	556	5.9
22	3493575	635	5.5
23	1912684	428	4.5
26	2307872	359	6.4
27	6230212	1253	5.0
28	4643423	815	5.7
29	5072401	953	5.3
30	3277867	526	6.2
31	115403	47	2.5
32	463681	165	2.8
33	6121876	1201	5.1
35	6319346	1330	4.8
37	2129589	463	4.6
38	3503286	835	4.2
