# Supplementary material for: Sequencing-based high throughput mutation detection in bread wheat
Source: BMC Genomics. 2015 Nov 17;16:962. doi: 10.1186/s12864-015-2112-1 (PMC4650848; doi:10.1186/s12864-015-2112-1)
Supplement: Additional file 8: — Is a table showing top and bottom strand barcode adapter sequences with barcode sequence in bold letters. (PDF 34 kb) [file 12864_2015_2112_MOESM8_ESM.pdf]

Additional data file 7 Top and bottom strand barcode adapter sequences with barcode sequence in bold letters

Plant ID	Top strand of barcode adapter
13	5 ACACTCTTTCCCTACACGACGCTCTTCCGATCTTGGC
14	5 ACACTCTTTCCCTACACGACGCTCTTCCGATCTCGAT
15	5 ACACTCTTTCCCTACACGACGCTCTTCCGATCTGAAT
16	5 ACACTCTTTCCCTACACGACGCTCTTCCGATCTTGCA
17	5 ACACTCTTTCCCTACACGACGCTCTTCCGATCTGCGT
18	5 ACACTCTTTCCCTACACGACGCTCTTCCGATCTCTTGA
19	5 ACACTCTTTCCCTACACGACGCTCTTCCGATCTAGCAT
20	5 ACACTCTTTCCCTACACGACGCTCTTCCGATCTCTAGC
21	5 ACACTCTTTCCCTACACGACGCTCTTCCGATCTACAAA
22	5 ACACTCTTTCCCTACACGACGCTCTTCCGATCTCGTCAA
23	5 ACACTCTTTCCCTACACGACGCTCTTCCGATCTCCAGCT
26	5 ACACTCTTTCCCTACACGACGCTCTTCCGATCTGAGAAT
27	5 ACACTCTTTCCCTACACGACGCTCTTCCGATCTGAGATA
28	5 ACACTCTTTCCCTACACGACGCTCTTCCGATCTCCACAA
29	5 ACACTCTTTCCCTACACGACGCTCTTCCGATCTTAGGAA
30	5 ACACTCTTTCCCTACACGACGCTCTTCCGATCTAACGCCT
31	5 ACACTCTTTCCCTACACGACGCTCTTCCGATCTCTTGCTT
32	5 ACACTCTTTCCCTACACGACGCTCTTCCGATCTAATATGC
33	5 ACACTCTTTCCCTACACGACGCTCTTCCGATCTGAATTCA
35	5 ACACTCTTTCCCTACACGACGCTCTTCCGATCTGGACCTA
37	5 ACACTCTTTCCCTACACGACGCTCTTCCGATCTGTCGATT
38	5 ACACTCTTTCCCTACACGACGCTCTTCCGATCTAAGGATGC
Indian	5 ACACTCTTTCCCTACACGACGCTCTTCCGATCTTAGCATGC
Indian	5 ACACTCTTTCCCTACACGACGCTCTTCCGATCTCGCCTTAT
	Bottom strand of barcode adapter
13	5 CWGGCCAAGATCGGAAGAGCGTCGTGTAGGGAAAGAGTGT
14	5 CWGATCGAGATCGGAAGAGCGTCGTGTAGGGAAAGAGTGT
15	5 CWGATTCAGATCGGAAGAGCGTCGTGTAGGGAAAGAGTGT
16	5 CWGTGCAAGATCGGAAGAGCGTCGTGTAGGGAAAGAGTGT
17	5 CWGACGCAGATCGGAAGAGCGTCGTGTAGGGAAAGAGTGT
18	5 CWGTCAAGAGATCGGAAGAGCGTCGTGTAGGGAAAGAGTGT
19	5 CWGATGCTAGATCGGAAGAGCGTCGTGTAGGGAAAGAGTGT
20	5 CWGGCTAGAGATCGGAAGAGCGTCGTGTAGGGAAAGAGTGT
21	5 CWGTTTGTAGATCGGAAGAGCGTCGTGTAGGGAAAGAGTGT
22	5 CWGTTGACGAGATCGGAAGAGCGTCGTGTAGGGAAAGAGTGT
23	5 CWGAGCTGGAGATCGGAAGAGCGTCGTGTAGGGAAAGAGTGT
26	5 CWGATTCTCAGATCGGAAGAGCGTCGTGTAGGGAAAGAGTGT
27	5 CWGTATCTCAGATCGGAAGAGCGTCGTGTAGGGAAAGAGTGT
28	5 CWGTTGTGGAGATCGGAAGAGCGTCGTGTAGGGAAAGAGTGT
29	5 CWGTTCCTAAGATCGGAAGAGCGTCGTGTAGGGAAAGAGTGT
30	5 CWGAGGCGTTAGATCGGAAGAGCGTCGTGTAGGGAAAGAGTGT
31	5 CWGAAGCAAGAGATCGGAAGAGCGTCGTGTAGGGAAAGAGTGT
32	5 CWGGCATTATTAGATCGGAAGAGCGTCGTGTAGGGAAAGAGTGT
33	5 CWGTGAATTCAGATCGGAAGAGCGTCGTGTAGGGAAAGAGTGT

35	5 CWGTAGGTCCAGATCGGAAGAGCGTCGTGTAGGGAAAGAGTGT
37	5 CWGAATCGACAGATCGGAAGAGCGTCGTGTAGGGAAAGAGTGT
38	5 CWGGCATCCTTAGATCGGAAGAGCGTCGTGTAGGGAAAGAGTGT
Indian	5 CWGGCATGCTAAGATCGGAAGAGCGTCGTGTAGGGAAAGAGTGT
Indian	5 CWGATAAGGCGAGATCGGAAGAGCGTCGTGTAGGGAAAGAGTGT
