# Supplementary material for: Sequencing-based high throughput mutation detection in bread wheat
Source: BMC Genomics. 2015 Nov 17;16:962. doi: 10.1186/s12864-015-2112-1 (PMC4650848; doi:10.1186/s12864-015-2112-1)
Supplement: Additional file 9: — Is a figure showing quality check for Ape KI enzyme cut genomic DNA library of hexaploid wheat on a DNA analyzer. (PDF 71 kb) [file 12864_2015_2112_MOESM9_ESM.pdf]

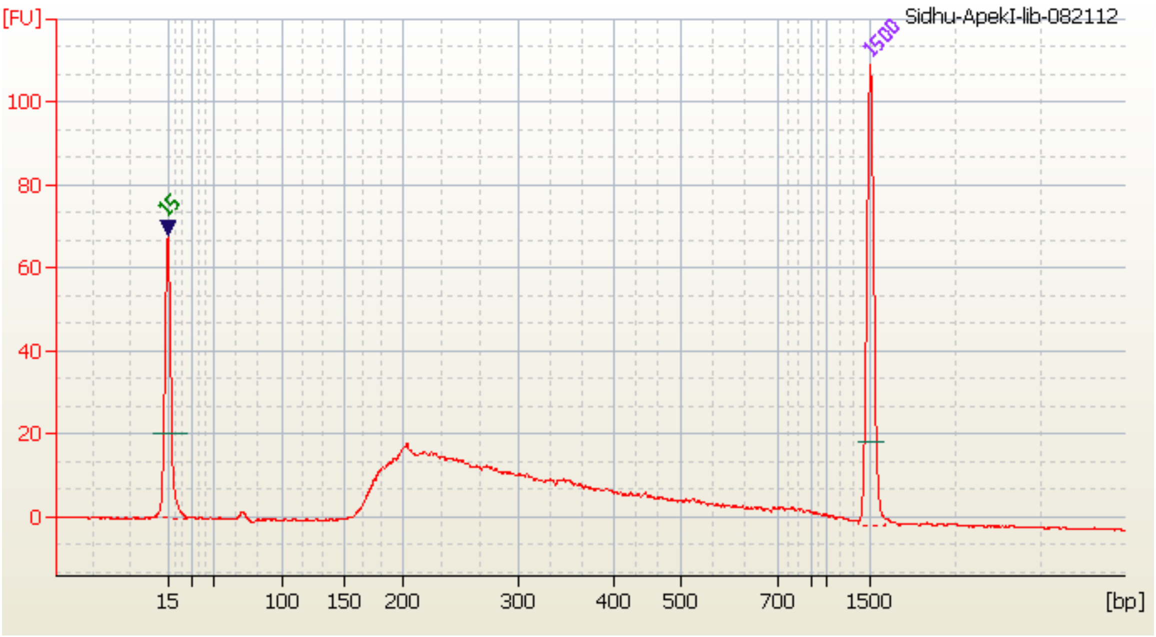

Additional data file 8 Quality check for *Ape*KI enzyme cut genomic DNA library of hexaploid wheat on a DNA analyzer. This library was sequenced since it gave an appropriate size and a very small adapter dimer peak.
